# Human TAU^P301L^ overexpression results in TAU hyperphosphorylation without neurofibrillary tangles in adult zebrafish brain

**DOI:** 10.1038/s41598-017-13311-5

**Published:** 2017-10-11

**Authors:** Mehmet I. Cosacak, Prabesh Bhattarai, Ledio Bocova, Tim Dzewas, Violeta Mashkaryan, Christos Papadimitriou, Kerstin Brandt, Heike Hollak, Christopher L. Antos, Caghan Kizil

**Affiliations:** 1 German Center for Neurodegenerative Diseases (DZNE), Arnoldstrasse 18, 01307 Dresden, Germany; 2 Center for Regenerative Therapies Dresden (CRTD), TU Dresden, Fetscherstrasse 105, 01307 Dresden, Germany; 3 School of Life Sciences and Technology, ShanghaiTech University, 100 Haike Road, Shanghai, China

## Abstract

Microtubule-associated TAU protein is a pathological hallmark in Alzheimer’s disease (AD), where hyperphosphorylation of TAU generates neurofibrillary tangles. To investigate the effects of TAU in a regenerative adult vertebrate brain system, we generated a cre/lox-based transgenic model of zebrafish that chronically expresses human TAU^P301L^, which is a variant of human TAU protein that forms neurofibrillary tangles in mouse models and humans. Interestingly, we found that although chronic and abundant expression of TAU^P301L^ starting from early embryonic development led to hyperphosphorylation, TAU^P301L^ did not form oligomers and neurofibrillary tangles, and did not cause elevated apoptosis and microglial activation, which are classical symptoms of tauopathies in mammals. Additionally, TAU^P301L^ neither increased neural stem cell proliferation nor activated the expression of regenerative factor Interleukin-4, indicating that TAU^P301L^ toxicity is prevented in the adult zebrafish brain. By combining TAU^P301L^ expression with our established Aβ42 toxicity model, we found that Aβ42 ceases to initiate neurofibrillary tangle formation by TAU^P301L^, and TAU^P301L^ does not exacerbate the toxicity of Aβ42. Therefore, our results propose a cellular mechanism that protects the adult zebrafish brain against tauopathies, and our model can be used to understand how TAU toxicity can be prevented in humans.

## Introduction

Accumulation of microtubule-associated TAU protein is a pathological hallmark of Alzheimer’s disease (AD) and frontotemporal dementia (FTD)^1–4^. TAU^P301L^ mutation leads to aggressive TAU aggregation and neurofibrillary tangle (NFT) formation in animal models of FTD^3–6^, whereas in AD, Aβ42 is believed to cause hyperphosphorylation of TAU, and subsequent aggregation^7–9^. In animal models of Tauopathies, motor deficits and neurodegeneration were observed^10–13^. Since Tauopathies are also seen in AD, transgenic models aiming to recapitulate the main symptoms of AD brains were generated using known familial mutations of AD and FTD, such as 3xTg^14^, TauPS2APP^15^, Tg2576^16^, PLB1-triple^17^, and rTg21221^18^. Although these models mimic the pathological features of the human disease, the proposed causative link between Aβ42 and TAU aggregation is still to be clarified^19–21^. Majority of the studies suggested a link between TAU toxicity and Amyloid aggregation^16,22–27^. However, there are also conflicting studies where Amyloid toxicity is not dependent on TAU^28–30^, and a recent study suggested that TAU might even ameliorate Amyloid toxicity^31^, indicating that the effects of Amyloid toxicity on TAU-dependent tangle formation, and the role of TAU tangles on exacerbating the Amyloid toxicity is still to be clarified. Therefore, new animal models where the effects of Aβ42 and TAU can be addressed independently and in combination to increase our understanding of the relationship between Aβ42 and TAU.

Neurodegenerative diseases cause a progressive deterioration of neuronal circuits and pronounced hampering of the stem cell proliferation^32^. Therefore, a plausible way of circumventing these diseases is to design regenerative therapy options where neurons would survive the toxicity burden exerted by TAU and Aβ42, but also the neural stem cells would enhance their proliferation to supply more cells that could be replenishing the function of the lost ones^33^. Mammalian models are excellent to recapitulate the pathophysiology of neurodegenerative diseases, however, since mammals are poorly regenerating, basic and translational questions regarding the regenerative capacity cannot be addressed well. Zebrafish, as a vertebrate serves as an excellent tool for questions concerning regeneration because of its high regenerative ability.

In zebrafish, several Tauopathy models were generated before. Transient expression of human TAU fused to GFP under gata2 promoter was shown to cause TAU phosphorylation in zebrafish larvae using Western blot analyses^34^. Another study using transient expression of human TAU-GFP under neuronal HuC/D promoter has also shown that TAU can be hyperphosphorylated^35^. The first stable TAU transgenic was generated by expressing TAU (0N4R) under the control of enolase promoter^36^. TAU was expressed in adult fish brain; however, no phenotypic characterization was documented. The best-characterized zebrafish TAU model so far is Gal4/UAS-mediated expression of human TAU^P301L^ under the control of HuC/D promoter^37^. This line shows motor neuron degeneration, hyperphosphorylation of TAU and tangle formation in the spinal cord. However, studies in this line only focused on the embryonic to juvenile spinal cord but not the brain. A recent study used a new allele of TAU with a mutation causing A152T conversion and showed hyperphosphorylation of TAU, motor neuron degeneration in the spinal cord, and cell death in larval zebrafish retina^38^. Therefore, these zebrafish models expressing human TAU have documented hyperphosphorylation of TAU and tauopathy in the spinal cord and the retina; however, the effects of TAU in the brain are understudied and therefore the effects are still unknown. In our study, we developed two conditional transgenic models of zebrafish, which expressed human TAU^P301L^ chronically in radial glial cells and the neurons derived from these progenitors in early embryonic development (with her4.1 promoter) and directly in neurons (with neural beta tubulin promoter). Expression of human TAU^P301L^ in adult zebrafish brain showed that human TAU^P301L^ can be phosphorylated, but neither forms any aggregates nor causes neurodegeneration on its own. Additionally, by combining the transgenic model of TAU^P301L^ expression with our recent Amyloid toxicity model^39,40^, we showed that Amyloid toxicity does not cause TAU aggregation, and TAU^P301L^ expression does not exacerbate the effects of Amyloid toxicity and AD-like neurodegeneration in adult zebrafish brain. Our results indicate that in adult zebrafish brain, human TAU^P301L^ can be phosphorylated but does not form aggregates and does not cause neurodegeneration, and Amyloid toxicity does not elicit Tauopathies.

## Results

### Conditional transgenic cassettes lead to strong neuronal expression of of TAUP301L in zebrafish brain

**Figure 1:**
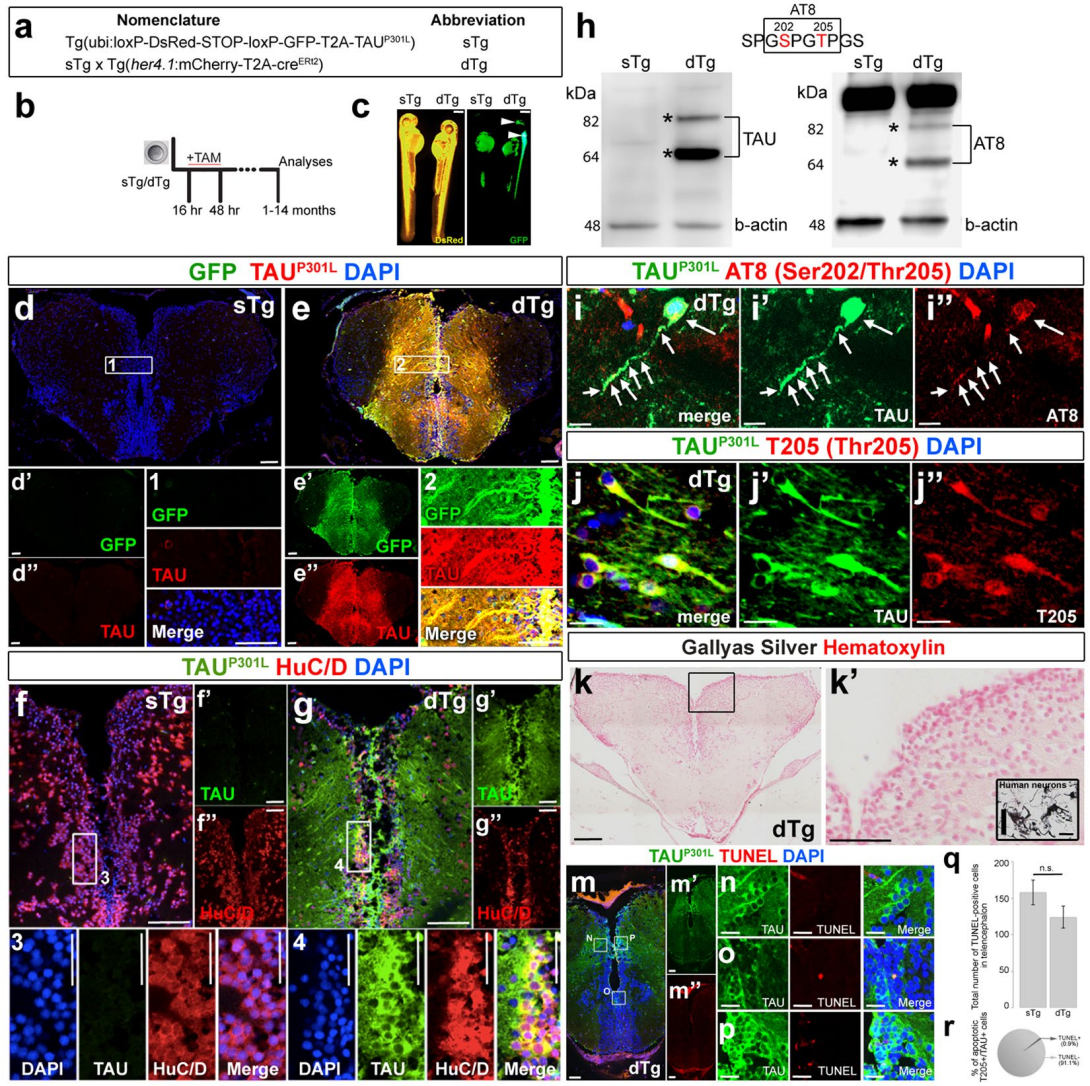
(**a**) Naming of transgenic constructs: sTg – effector transgenic, dTg – double transgenic. (**b**) Scheme for timing of recombination and analyses. (**c**) sTg and dTg animals (5 days post fertilization) treated with tamoxifen. DsRed expression indicates the presence of the effector cassette. Note the recombination in the central nervous system by GFP expression. (**d**) Immunohistochemistry (IHC) for GFP and TAU^P301L^ on coronal sections of telencephalon of a 6 month-old sTg animal. Single channel images of the whole section for GFP (d’) and TAU (d”). (1) is the enlarged view of the inset in d. (**e**) IHC for GFP and TAU^P301L^ on coronal sections of telencephalon of a 6-month old dTg animal. Single channel images of the whole section for GFP (e’) and TAU (e”). (2) is the enlarged view of the inset in f. (**f**) IHC for HuC/D and TAU^P301L^ on coronal sections of telencephalon of an sTg animal. Single channel images of the whole section for TAU (f’) and HuC/D (f”). (3) is the enlarged view of the inset in f. (**g**) IHC for HuC/D and TAU^P301L^ on coronal sections of telencephalon of a dTg animal. Single channel images of the whole section for TAU (g’) and HuC/D (g”). (4) is the enlarged view of the inset in g. (**h**) Western blot analyses for expression of TAU^P301L^ (left) and hyperphosphorylated TAU^P301L^ (right) in telencephalon. Beta actin is used as a loading control. (**i**) IHC for GFP and AT8 in a dTg animal. Individual channels are shown for GFP (i’) and AT8 (i”). Arrows represent the cytoplasmis signal. (**j**) IHC for TAU^P301L^ and T205 in a dTg animal. Individual channels are shown for TAU^P301L^ (j’) and T205 (j”). (**k**) Gallyas silver (black) and Hematoxylin (pink) staining in telencephalon of a dTg animal. (k’) Enlarged region in k’. Note the absence of Gallyas silver-positive cells. (**l**) Positive control for Gallyas silver staining in human neurons treated with Amyloid, showing neurofibrillary tangles. (**m**) Immunohistochemistry for TAU^P301L^ (green) combined with TUNEL detection of apoptotic cells (red) in recombined 6 months old dTg animals. Individual fluorescent channels are shown in m’ and m”. (**n**–**p**) Higher magnification images from the insets in m. (**q**) Quantification of TUNEL-positive cells in the telencephalon in sTg and dTg animals. (**r**) Quantification of apoptotic cells containing hyperphosphorylated TAU^P301L^ (T205-positive). Values represent mean ± s.e.m. *p < 0.05, **p < 0.01, ***p < 0.005. Scale bars equal 10 μm (**i**–j’) and 50 μm elsewhere. n = 6 fish and > 30 histological sections for every staining. Larvae are 5 days old, and adult animals are 6 months old.

To investigate the chronic effects of human TAU^P301L^ expression in zebrafish brain, we generated a cre/lox-based conditional double-transgenic zebrafish that expresses TAU^P301L^. The spatial control cassette drives the expression of inducible cre recombinase (creERT2) under the control of *her4*.*1* promoter, which is active in neural stem/progenitor cells with radial glial identity (Fig. 1a, Tg(her4.1:mCherry-T2A-creERT2)). We specifically chose *her4*.*1* promoter because we hypothesized that if the expression of TAU^P301L^ started from the stem cell stage, all neurons produced by those stem cells after the recombination would express the protein. In contrast to conditional expression systems that use mature neuronal promoters such as HuC/D^41^, enolase^36^ or neural beta tubulin (NBT)^42^, using a stem cell promoter would increase the number of neurons expressing the transgene throughout the life of the fish. Additionally, since in familial forms of neurodegenerative diseases, mutant proteins are expressed in all cells – including the stem cells - of the patients during development, our approach would also help to address the effects of TAU^P301L^ in neural stem cells of zebrafish brain in a way reminiscent of familial forms of Tauopathies in humans.

The effector cassette is responsive to cre and expresses TAU^P301L^ under the ubiquitous promoter after recombination (Fig. 1a, Tg(ubi:loxP-DsRed-STOP-loxP-GFP-T2A-TAU^P301L^). From here on, Tg(ubi:loxP-DsRed-STOP-loxP-GFP-T2A-TAU^P301L^) will be named as sTg, double transgenic (Tg(ubi:loxP-DsRed-STOP-loxP-GFP-T2A-TAU^P301L^) and Tg(her4.1:mCherry-T2A-creERT2) will be denoted as dTg). Since cre activity is induced by addition of Tamoxifen (TAM), we treated sTg and dTg animals with TAM from 16 hours to 48 hours post fertilization in order to recombine all radial glial cells (RGCs), and analyzed the adult animals at 1, 3, and 6 months post fertilization (Fig. 1b). While the recombination does not take place without TAM, after TAM treatment at 48 hours post fertilization dTg animals show GFP expression along the whole rostrocaudal axis of the central nervous system (Fig. 1c).

To determine how widespread TAU^P301L^ is expressed by using *her4*.*1* promoter, we performed immunostainings for GFP and TAU^P301L^ on sagittal sections of the adult zebrafish brain (Supplementary Fig. 1). We observed a strong and reproducible expression of TAU^P301L^ in adult zebrafish brain from olfactory bulb to the hindbrain (Supplementary Fig. 1 insets 1–9). These results indicate that TAU^P301L^ is successfully expressed throughout the entire axis of zebrafish brain using a stem cell promoter. In order to further investigate whether the use of *her4*.*1* promoter is appropriate to express TAU in maximum number of neurons, we expressed TAU by using the same recombination paradigm before but using a cre-driver line expressing cre recombinase under the control of neural beta-tubulin promoter (NBT): Tg(nbt:mCherry-T2A-creERT2) (designated hereafter as nTg). By performing recombination after crossing nTg to sTg, we found that nTg is leading to transgene expression along the rostrocaudal axis of the fish brain, and, compared to *her4*.*1*-driven TAU expression, *nbt* promoter also drives expression of the transgene in a similarly widespread manner (Supplementary Fig. 2), suggesting that *her4*.*1* promoter can be used to target neurons in the adult zebrafish brain by cre-mediated recombination.

Based on the known pathological locations of TAU in human brains, we focused on the forebrain for the rest of our analyses. By performing immunostainings for GFP and TAU^P301L^ on coronal sections of the zebrafish forebrain, we determined that compared to control animals (sTg), dTg animals show a specific pattern of recombination, which colocalizes with TAU^P301L^ expression in the telencephalon (Fig. 1d–e”; Supplementary Fig. 3). To investigate whether TAU^P301L^ is expressed in neurons, we performed immunostainings for TAU^P301L^ and neuronal marker HuC/D in sTg (Fig. 1f–f’) and dTg animals (Fig. 1g–g”). We observed that neurons express TAU^P301L^ (Fig. 1g4). To determine the percentage of neurons in the pallium that express TAU^P301L^, we quantified the number of cells that co-localize HuC/D and TAU^P301L^ (Supplementary Fig. 4). We found that TAU^P301L^ is expressed in 91.8% % of the neurons (Supplementary Fig. 4), indicating that in our model, majority of the neurons in the forebrain express TAU^P301L^.

### TAU^P301L^ is hyperphosphorylated in adult zebrafish brain

One of the hallmarks of Tauopathies in human brains is hyperphosphorylation of TAU and formation of neurofibrillary tangles, which can be detected by histological stainings. To analyze whether chronic expression of human TAU^P301L^ in adult zebrafish brain leads to hyperphosphorylation and tangle formation, we analyzed the immunoreactivity of the adult fish brain to AT8, an indicator of late stage Tau hyperphosphorylation at the Serine-202 and Threonine-205 (Fig. 1h). Western blot analyses showed that human TAU^P301L^ is specifically expressed only in dTg but not sTg animals, and when compared to the beta-actin loading control, the expression is strong and abundant (Fig. 1h). We also found that TAU^P301L^ leads to AT8 immunoreactivity (~70 kDa expected size), which is suggestive of TAU^P301L^ hyperphoshorylation (Fig. 1h). The band around 82 kDa is the fusion of GFP and TAU in the cre-effector cassette, and results from inefficient cleavage of t2a peptide in approximately 8% of the cases as documented before^43^. The strong band above 82 kDa is a non-specific recognition of AT8 in zebrafish in the nucleus (Supplementary Fig. 5). By performing quantification of the relative intensities of detected bands, we found that 3.8 ± 1.4% of the total TAU is AT8-positive in adult zebrafish brain. To confirm the results from western blot analyses, we performed immunohistochemical stainings for AT8 and TAU^P301L^ (Fig. 1i–i”). We observed that a small portion of the TAU^P301L^-positive cells display AT8 immunoreactivity in the cytoplasm (Fig. 1i–i”). However, since AT8 staining leads to a nuclear background staining in adult zebrafish brain and therefore determining the cytoplasmic staining in adult brain is difficult (Supplementary Fig. 5). To further verify the phosphorylation status of TAU, we performed immunohistochemical staining with an antibody that specifically recognizes phosphorylation of Threonine-205 (T205) (Fig. 1j–j”’). We found that this staining does not cause background signals (Fig. 1j–j”), and approximately 76.1% of the TAU^P301L^-positive cells are also positive for T205 phosphorylation (Supplementary Fig. 6). These results indicate that chronic expression of TAU^P301L^ can cause hyperphosyphorylation in adult zebrafish brain. We also analyzed the early phosphorylation of TAU by immunohistochemistry and western blot for AT180 (Threonine-231 and Serine-235) (Supplementary Fig. 7) and AT270 (Threonine-181) (Supplementary Fig. 7). We found that AT180 and AT270 give strong non-specific cross-reactivity in zebrafish in immunohistochemistry (Supplementary Fig. 7a,b”’, Supplementary Fig. 8a,b”’), and AT270 additionally show non-specific signal in western blots (Supplementary Fig. 8c), indicating that these antibodies cannot be reliably used. AT180, however, shows a specific band of phosphorylated TAU in dTg animals (Supplementary Fig. 7c), confirming the phosphorylation determined by AT8 and T205 antibodies. In overall, these results indicate that TAU^P301L^ is hyperphosphorylated in adult zebrafish brain.

Hyperphosphorylation of TAU^P301L^ is known to cause neurofibrillary tangle (NFT) formation^1,2^, and to test whether chronic expression of human TAU^P301L^ would lead to NFTs, we histologically stained coronal sections of adult zebrafish telencephalon with Gallyas silver (Fig. 1k,k’). We did not observe any tangle formation, whereas a positive control from human neurons treated with Amyloid-β42 gave positive signal for Gallyas silver (Fig. 1l), indicating that despite its hyperphosphorylation, human TAU^P301L^ does not form NFTs in adult zebrafish brain.

**Figure 2:**
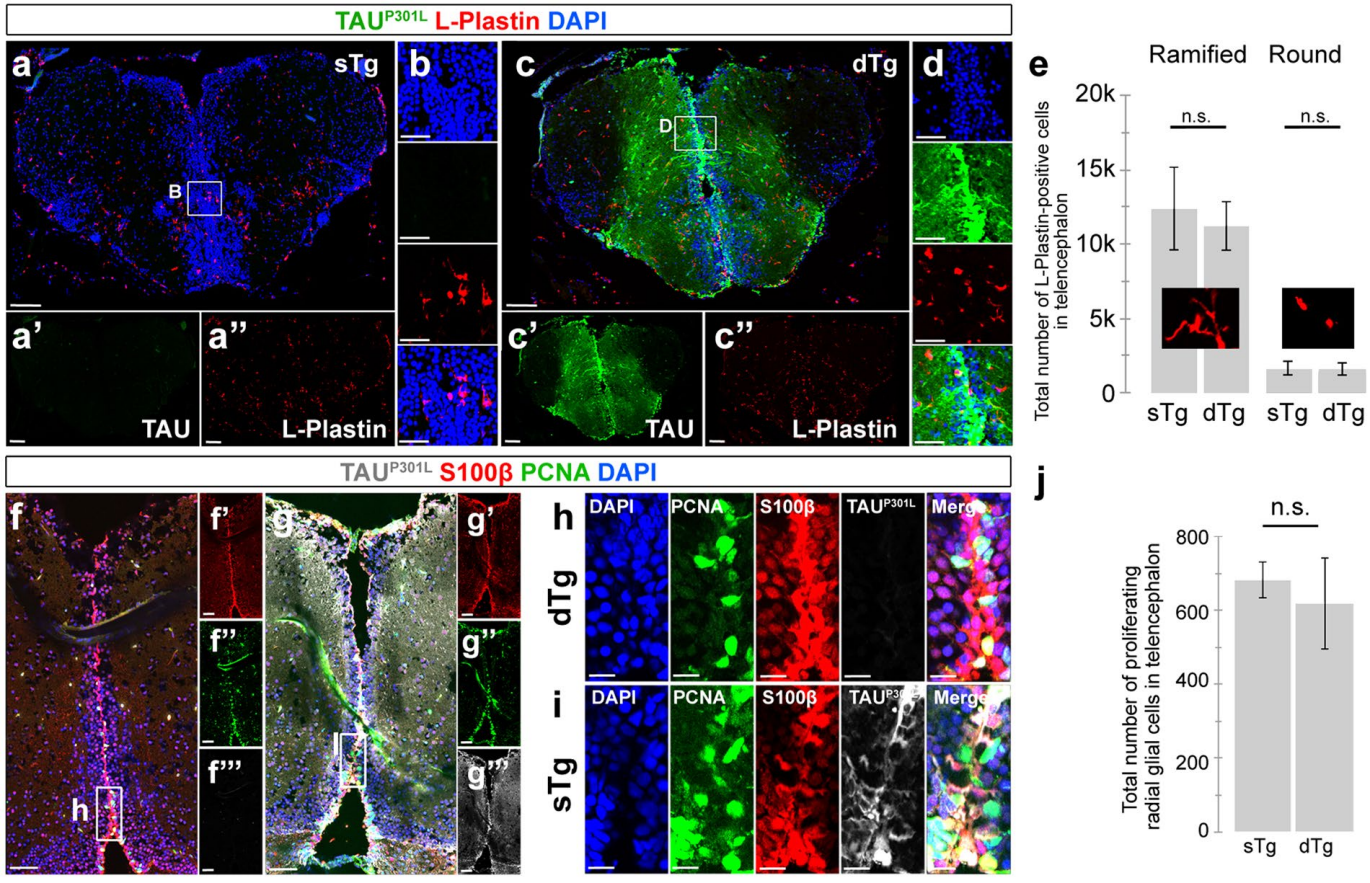
(**a**) Immunohistochemistry (IHC) for L-Plastin (red) and TAU^P301L^ (green) on coronal sections of telencephalon of a 6 month-old sTg animal. Single channel images of the whole section for TAU^P301L^ (a’) and L-Plastin (a”). (**b**) The enlarged view of the inset in a. (**c**) IHC for L-Plastin (red) and TAU^P301L^ (green) on coronal sections of telencephalon of a 6-month old dTg animal. Single channel images of the whole section for TAU^P301L^ (c’) and L-Plastin (c”). (**d**) The enlarged view of the inset in c. (**e**) Quantification of round and ramified L-Plastin-positive cells in the telencephalon in sTg and dTg animals. (**f**) Immunohistochemistry (IHC) for S100β (red), PCNA (green) and TAU^P301L^ (white) on coronal sections of telencephalon of a 6-month old sTg animal. Single channel images of the whole section for S100β (F’), PCNA (f”), and TAU^P301L^ (f”’). (**g**) IHC for S100β (red), PCNA (green) and TAU^P301L^ (white) on coronal sections of telencephalon of a 6-month old dTg animal. Single channel images of the whole section for S100β (g’), PCNA (g”), and TAU^P301L^ (g”’). (**h**) The enlarged view of the inset in f. (**i**) The enlarged view of the inset in g. (**j**) Quantification of the total number of proliferating glial cells in the telencephalon of sTg and dTg animals. Values represent mean ± s.e.m. *p < 0.05, **p < 0.01, ***p < 0.005. Scale bars equal 50 μm (**a**–g”’) and 20 μm (**h**,**i**). n = 7 fish and > 30 histological sections for every staining. All animals are 6 months old.

We hypothesized that if NFTs – as the toxic outcome of TAU – do not form in adult zebrafish brain, the pathological outcomes of neurodegeneration would not manifest in dTg animals. To test this hypothesis, we investigated the levels of apoptosis (Fig. 1m–q), microglia/macrophage activation as an indication of chronic inflammation (Fig. 2a–e) and proliferation of radial glial cells (RGCs) as a standard readout after neuronal loss in adult zebrafish brain^39,44,45^ (Fig. 2f–j).

### Phosphorylated TAU^P301L^ does not cause cell death, inflammation and stem cell activation in adult zebrafish brain

To determine whether the cells where TAU is hyperphosphorylated undergo apoptosis, we performed TUNEL staining and found that the overall levels of cell death in dTg animals is not significantly different than sTg animals (Fig. 1q), while our previously established AD model of adult zebrafish brain shows extensive cell death (Supplementary Fig. 9), which is also confirmed by unchanged relative expression levels of apoptotic regulator Caspase 3 (Supplementary Fig. 9i). We also observed that only a minor fraction of T205-positive cells containing phosphorylated-TAU are TUNEL positive (Fig. 1r). These results indicate that TAU^P301L^ expression does not lead to cell death in adult zebrafish brain.

Tauopathies cause chronic inflammation by significantly inducing the numbers of macrophages/microglia as well as favoring for their activation state^46^. To test if chronic expression of human TAU^P301L^ in adult zebrafish brain would alter the microglial dynamics, we performed immunostaining for L-Plastin, a marker for microglia and macrophages in adult zebrafish brain (Fig. 2a–d). Compared to sTg, dTg animals did not show a significant difference in the overall number and activation state of the microglia, as the number of ramified and round L-Plastin-positive cells remains unchanged (Fig. 2d). This result indicates that chronic expression of human TAU^P301L^ does not induce chronic activation of microglia in adult zebrafish brain, and this suggests that chronic inflammation does not take place.

Zebrafish has an extensive regenerative ability in its brain, and loss of neurons causes radial glial cells to respond by increasing their proliferation^39,44,45,47–53^. To test if chronic expression of TAU^P301L^ would induce the proliferation of neuronal progenitors, we performed immunostaining for S100β (a progenitor cell marker) and PCNA (proliferation marker) in sTg and dTg animals (Fig. 2f–i). Compared to sTg animals, chronic expression of TAU^P301L^ did not alter the level of proliferation of radial glial cells (Fig. 2j). Additionally, similar to *her4*.*1*, *nbt*-driven TAU did not alter neural stem cell proliferation (S100β-PCNA stainings) or microglial activation in adult zebrafish brain (L-Plastin stainings) (Supplementary Fig. 2). These results indicate that despite the presence and hyperphosphorylation of human TAU^P301L^, zebrafish brain does not show any pathological hallmarks of Tauopathies, and major pathological hallmarks - cell death, inflammation and cell proliferation - are not affected. We also confirmed these findings by detecting the relative mRNA expression levels of pro-inflammatory cytokines *tnfa*, *ifng*, *il1b*, *il6*, and *il12a* (Supplementary Fig. 10), and observed that these genes do not change their expression levels.

### Phosphorylated TAU^P301L^ neither exacerbates the toxicity of Aβ42 nor initiates regeneration programs in adult zebrafish brain

**Figure 3:**
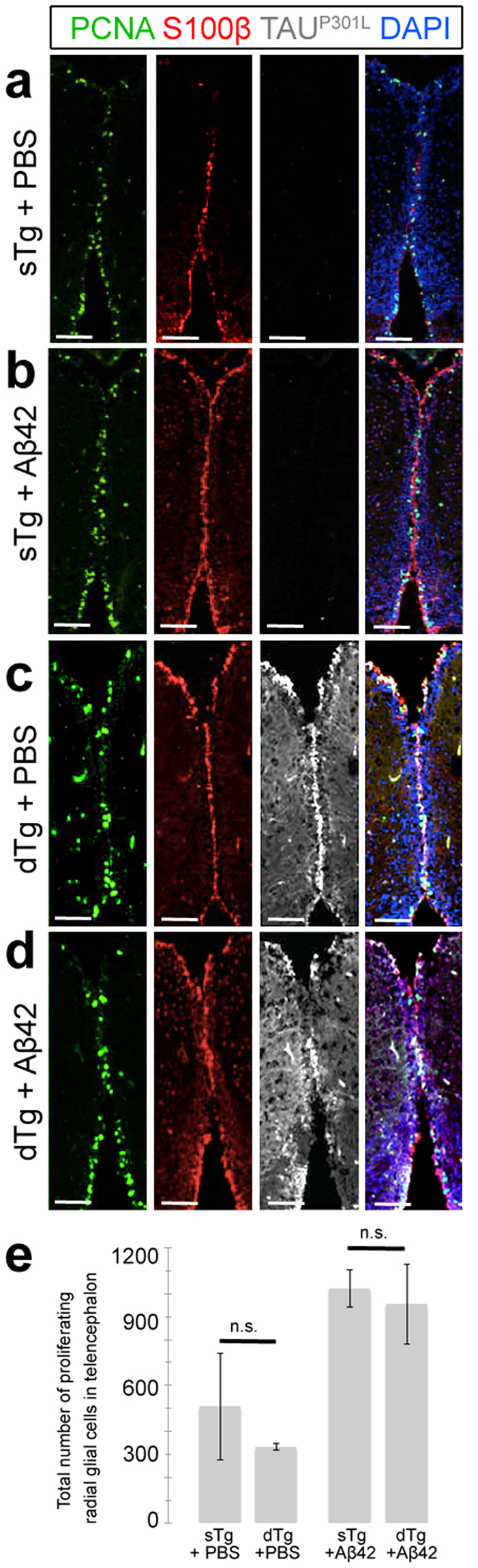
Immunohistochemistry for PCNA (green), S100β (red), and TAU^P301L^ (white) on coronal sections of telencephalon of a 6 month-old sTg animal injected with PBS (**a**), sTg animal injected with Aβ42 (**b**), dTg animal injected with PBS (**c**), and dTg animal injected with Aβ42 (**d**). (**e**) Quantification of the total number of proliferating radial glial cells in the telencephalon of sTg and dTg animals injected with PBS or Aβ42. Values represent mean ± s.e.m. *p < 0.05, **p < 0.01, ***p < 0.005. Scale bars equal 100 μm. n = 5 fish and > 20 histological sections for every staining. All animals are 6 months old.

In Alzheimer’s mouse models and cultures of human neurons, Amyloidβ42 (Aβ42) deposition leads to hyperphoyphorylated TAU and NFT formation^54–56^, and Tauopathies are suggested to mediate the Amyloid toxicity^9,18,56–58^. We have recently generated an Aβ42 toxicity model in adult zebrafish brain, where Aβ42 aggregation leads to cell death, inflammation and induction of radial glial cell proliferation and neurogenesis^39^. We hypothesized that by combining the AD model of adult zebrafish brain to the transgenic expression of human TAU^P301L^ would help determining if chronic expression of TAU^P301L^ would exacerbate the effects of Aβ42. Therefore, we analyzed the radial glial cell proliferation in sTg and dTg animals injected with PBS or Aβ42 as readout of pathology and regenerative response (Fig. 3a–e). In both sTg and dTg animals, injection of Aβ42 increased the cell proliferation to the same degree (Fig. 3e), and Aβ42 aggregation did not cause NFT formation (data not shown), indicating that TAU^P301L^ does not exacerbate the pathological burden of Aβ42 in zebrafish.

**Figure 4:**
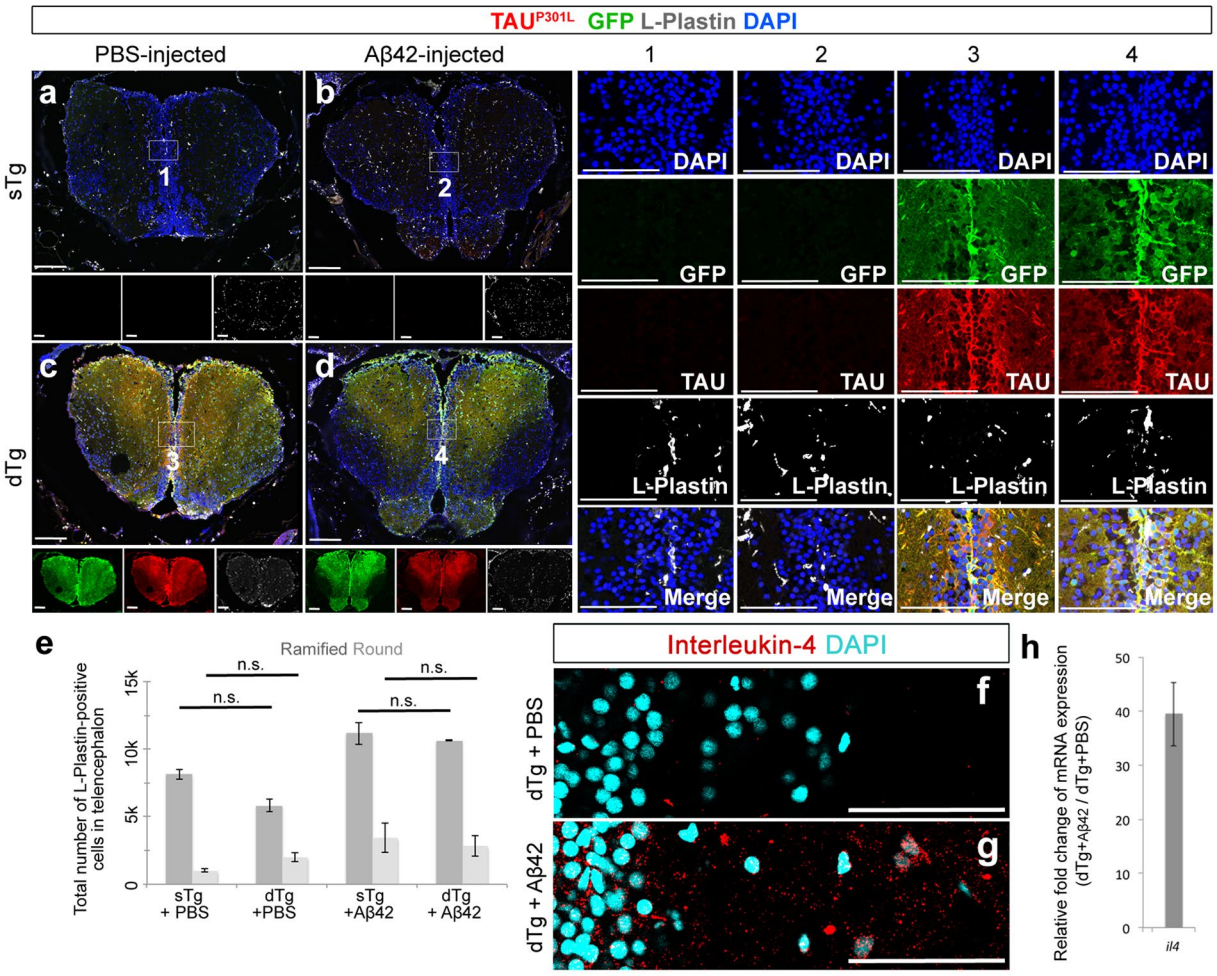
(**a**) Immunohistochemistry (IHC) for TAU^P301L^ (red), GFP (green) and L-Plastin (white) on coronal sections of telencephalon of a 6 month-old sTg animal injected with PBS (**a**), sTg animal injected with Aβ42 (**b**), dTg animal injected with PBS (**c**), and dTg animal injected with Aβ42 (**d**). Insets below the panels show individual fluorescent channels for TAU^P301L^ (red), GFP (green) and L-Plastin (white). Columns 1–4 are single and merged images of the regions indicated in (**a–d**), respectively. (**e**) Quantification of the total number of ramified and round L-Plastin-positive cells in the telencephalon of sTg and dTg animals injected with PBS or Aβ42. (**f–g**) Immunohistochemistry for Interleukin-4 (red) on coronal sections of telencephalon of a 6-month old dTg animal injected with PBS (**f**) or with Aβ42 (**g**). (**h**) Relative change in the expression levels of *il4* after Aβ42 injection compared to control injection. Medial ventricular regions are shown. DAPI (cyan) marks the nuclei. Values represent mean ± s.e.m. *p < 0.05, **p < 0.01, ***p < 0.005. Scale bars equal 50 μm. n = 6 fish and > 30 histological sections for every staining. All animals are 6 months old.

A typical symptom of Amyloid toxicity is inflammation and activation of macrophages/microglia^59–61^. To determine whether TAU^P301L^ expression would alter the inflammatory outcome exerted by Aβ42, we performed immunostaining for L-Plastin – a marker for macrophages/microglia – in sTg and dTg TAU^P301L^ lines (Fig. 4a–d). We found that Aβ42 increases both the ramified and round macrophage/microglia in sTg animals as we have documented before^39^, and this increase is statistically not different in TAU^P301L^-expressing dTg animals (Fig. 4e). Additionally, sTg and dTg animals that are not injected with Aβ42 do not differ in the number and activation state of macrophages/microglia (Fig. 4e). These results suggest that chronic expression human of TAU^P301L^ does not change the number and activation state of the microglia/macrophages as determined by L-Plastin staining, and does not exacerbate the effects of Aβ42 in adult zebrafish brain, and interestingly despite the widespread expression of TAU in adult zebrafish brain (Supplementary Figs 1 and 2), Aβ42 does not cause NFT formation from the TAU expressed thereof.

In adult zebrafish brain, Aβ42 was shown to cause a specific neurodegeneration-induced regeneration response by activating the expression of Interleukin-4 (IL4) – a biomarker of neurodegeneration-induced regenerative response in adult zebrafish brain^39^. Since TAU^P301L^ expression and its hyperphosphorylation did not lead to changes in cell death and stem cell proliferation, we hypothesized that in our TAU model, we would not see the neurodegeneration induced IL4 expression. To test this hypothesis, we performed immunohistochemical staining for IL4 in dTg animals injected with PBS or Aβ42 (Fig. 4f,g). We found that IL4 is not expressed in dTg animals with PBS injection (Fig. 4f), whereas Aβ42 injection leads to induction of IL4 expression that is detected by both immunostaining (Fig. 4g) and relative mRNA expression levels (upregulation by 39.5 ± 5.9 folds) (Fig. 4g,h). These results support our previous findings that TAU^P301L^ hyperphosphorylation does not lead to neurodegeneration and subsequent regeneration response in adult zebrafish brain.

In several mouse models of TAU^P301L^, the phenotypes manifest in aged animals^62^. We have recently shown that aging exacerbates the neurodegeneration phenotypes in adult zebrafish brain^40^. Therefore, we hypothesized that chronic expression of TAU^P301L^ in older zebrafish could manifest Tauopathies that we did not observe in younger fish (1–6 months of age). To test this hypothesis, we analyzed TAU expression (Supplementary Fig. 11a–f) and microglia activation that is indicative of chronic inflammation (Supplementary Fig. 11g–l) in 14 month-old adult zebrafish brains. We observed that although TAU is expressed strongly at 14 month-old brains of dTg animals (Supplementary Fig. 11d–f), this does not cause macrophage/microglia activation (Supplementary Fig. 11g–m), indicating that the lack of Tauopathies in adult zebrafish brain is not due to the late manifestation as seen in mouse models.

### TAU^P301L^ is phosphorylated in zebrafish larvae yet causing no overt behavioral phenotypes

Our TAU model of adult zebrafish brain does not form neurofibrillary tangles despite phosphorylation of the TAU protein. Previously, phosphorylated TAU was documented in zebrafish spinal cord and these fish showed defective escape response at 2 days of development, yet they can regain all behavioral functions at 4 days of development and afterwards^37^ suggesting that zebrafish can effectively overcome TAU-mediated pathology. To investigate whether in our zebrafish lines TAU is expressed and phosphorylated in larval stages, we performed immunohistochemical staining for TAU and western blot analysis for phosphorylated TAU markers AT8, AT180, and T205 (Supplementary Fig. 12). We found that TAU is widely expressed in zebrafish larvae along the whole rostrocaudal axis of the central nervous system, and it is phosphorylated (Supplementary Fig. 12). By band intensity quantifications, we found that 91.4 ± 4.7% of total TAU is AT180-positive and 70.3 ± 4.7% of total TAU is AT8-positive in the whole larvae.

In order to examine whether the larval fish shows escape response deficits, we performed a behavioral assay for 2- and 4 day-old larvae as described before^37^ (Supplementary Videos 1 and 2). We observed that dTg larvae show normal escape behavior as in their non-transgenic siblings and sTg counterparts (Supplementary Videos 1 and 2), indicating that the Tauopathy in the spinal cord of the larvae is causing the behavioral deficits. These results recapitulate what has been documented before^37,63^.

### TAU^P301L^ does not form oligomers in adult zebrafish brain

Our results showed that while TAU^P301L^ is phosphorylated, neurofibrillary tangles do not form. We hypothesized that this could be because TAU^P301L^ might not be forming oligomers that are the precursors of tangles. In order to test this hypothesis, we performed immmunohistochemical staining with T22 antibody that detects TAU oligomers. We found that TAU^P301L^ does not form oligomers in adult zebrafish brain in the presence or absence of Amyloid-β42 (Supplementary Fig. 13). These results indicate that in adult zebrafish brain, despite being phosphorylated, TAU^P301L^ does not form oligomeric forms of TAU, and this could be the reason for absence of neurofibrillary tangles.

## Discussion

**Figure 5:**
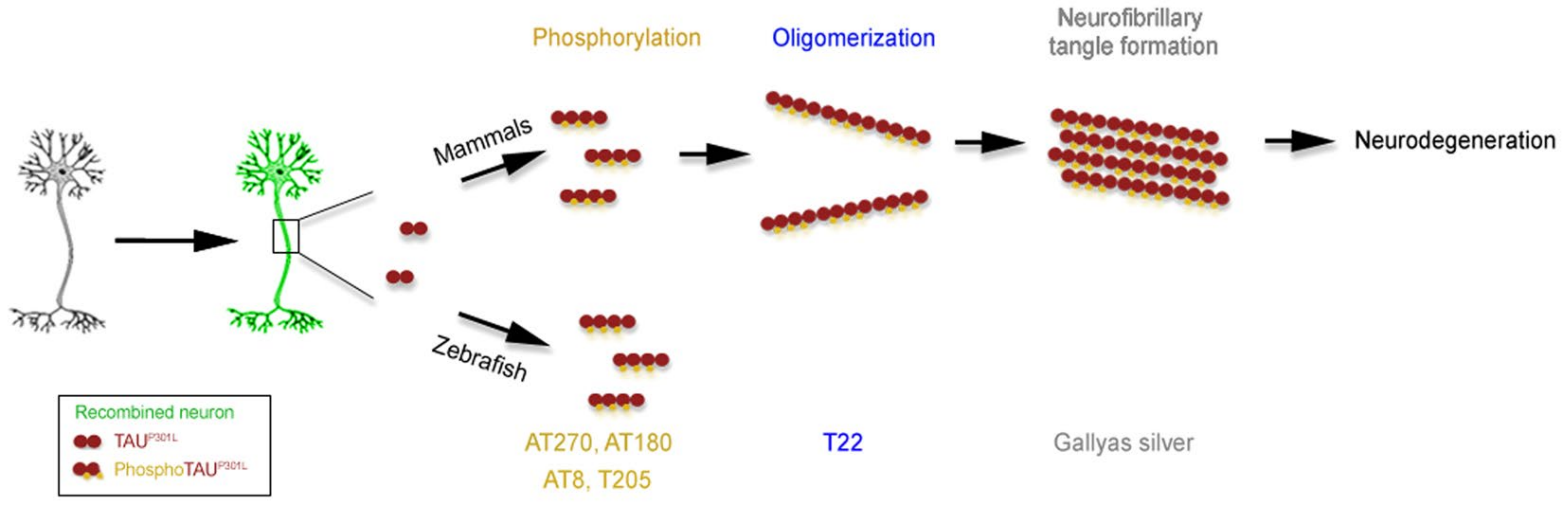
Schematic comparison of the effects of TAU in mammalian and zebrafish brain.

In this study, we generated two cre-lox-based conditional expression lines for adult zebrafish brain to study the effects of chronic expression of human TAU^P301L^ and its relation to Aβ42 toxicity. We found that although chronically expressed TAU is hyperphosphorylated, it does not form oligomers and neurofibrillary tangles, and hence does not cause neurodegeneration in the adult zebrafish brain (Fig. 5).

Previous reports of TAU in zebrafish used larval and juvenile animals^34,37,63^ or did not thoroughly investigate the adult phenotypes^36^. Our study is therefore the first study investigating TAU in a chronic expression model of adult zebrafish brain, where we found that human TAU^P301L^ was hyperphosphorylated as documented before in zebrafish^36,64^. However, hyperphosphorylation of TAU did not cause formation of oligomers and neurofibrillary tangles in adult zebrafish brain. We believe that this is not due to the inability of phosphorylated TAU to form neurofibrillary tangles *per se*, but because of a specific blockage of tangle formation in adult zebrafish brain.

The lack of tangle formation despite the presence of hyperphosphorylated form of TAU might be due to several reasons. First, zebrafish brain might not have the circuitries and neuronal subtypes that are susceptible to TAU aggregation in mammals, and therefore modeling Tauopathies in zebrafish might not be appropriate. We believe that this is not the case, because several studies have shown that adult zebrafish brain have analogous regions to the human brains^65,66^, and the majority of the neuronal subtypes present in our brains are existent in adult zebrafish brain^45,67–69^. Additionally, all the constituents of the machinery known to be required for TAU phosphorylation and aggregation is present in zebrafish^37,63^ as zebrafish proteome contains more than 80% of the proteins present in humans^70^ and the kinases that are implicated in TAU hyperphosphorylation (e.g.: GSK3β, Cdk5, MARK1 and CK1α^71^) are present in zebrafish, and are expressed (Supplementary Fig. 14). Second, the expression level of TAU is known to be a critical determinant of the aggregation dynamics^62^. In our experiments, we used ubiquitin promoter, which drives the expression of the downstream gene efficiently^72^. Also, our western blot results indicate that the levels of TAU are higher than the housekeeping gene beta-actin (Fig. 1h), and immunohistochemical stainings detect strong expression of TAU protein (Figs 1e, g and 2c). Based on these results, we believe that the lack of aggregation of TAU is not due to the levels of TAU protein. Third, our transgenic line may not be expressing TAU in the neurons that are susceptible to Tauopathies in humans. This is a challenging possibility, yet, as we determined by sagittal sections of the adult fish brain (Supplementary Figs 1 and 2), *her4*.*1* and *nbt* promoters can lead to TAU expression in the majority of the neurons in the adult fish brain, and it is very unlikely that such a widespread expression would not include the neurons of the forebrain that are susceptible to Tauopathies. Therefore, we hypothesize that the lack of neurofibrillary tangles in our model is not due to targeting the right cell types but due to an active protective mechanism that prevents phosphorylated TAU from forming tangles. This is an interesting option suggesting that zebrafish brain could serve as an *in vivo* model for experimentation on how Tauopathies can be prevented by using endogenous cellular programs. Further research on why zebrafish does not form Tauopathies in the brain while it does in the larval spinal cord^37,63^ will constitute an exciting basic and pre-clinical research line. For instance, prevention of TAU dephosphorylation by Okadaic acid was shown to increase hyperphosphorylated state of TAU and subsequently leading to neurofibrillary tangles^73^. When we injected Okadaic acid to TAU^P301L^-expressing adult zebrafish brains, we still did not observe neurofibrillary tangle formation (data not shown), suggesting a currently unknown cellular mechanisms that might be utilized to prevent phosphorylated TAU from forming tangles in human brains. We also found that in the larvae of our zebrafish model, TAU is expressed and hyperphosphorylated (Supplementary Fig. 12) but does not show any behavioral phenotypes such as escape response (Supplementary Videos 1 and 2). Additionally, adult dTg zebrafish do not show any swimming defects or escape response (data not shown) suggesting that TAU hyperphosphorylation does not cause NFT formation in larval and adult stages in our model. Thus, using our zebrafish TAU model as a tool to uncover putative protective mechanisms that prevent phosphorylated TAU from forming tangles will be of great interest to the medical field.

It is still controversial whether neuroinflammatory events lead to TAU phosphorylation or phosphorylated-TAU precedes neuroinflammation^46,57,74–76^. Our results suggest that phosphorylated-TAU is not sufficient to induce microglial activation by itself, and that aggregated forms of TAU might be required for this. We found that in our model, oligomeric TAU is not formed (Supplementary Fig. 13), suggesting that TAU phosphorylation might not be caused by immune cell activity and more toxic forms of TAU (such as oligomers) are required to elicit neuroinflammation by TAU. Our model can help to investigate this interesting hypothesis by analyzing the effects of TAU on various immune cell types, not necessarily confined to microglia, but also other immune cells involved in adaptive immune responses.

We recently established an Amyloid-β42 toxicity model in zebrafish, and observed that Aβ42 aggregation causes cell death, inflammation, synaptic degeneration, and memory deficits, which collectively lead to regenerative response including stem cell activation and neurogenesis^39,62^, signifying the role of neuro-immune cross talk for efficient regeneration response in contrast to mammals^77,78^. When we combined amyloid toxicity model with chronic genetic expression of human TAU^P301L^, we found that TAU expression does not exacerbate the Amyloid phenotypes, and Amyloid toxicity does not cause TAU-mediated neurofibrillary tangle formation. Additionally, unlike Aβ42, TAU^P301L^ does not induce the expression of neurodegeneration-induced factor Interleukin-4^39^ (Fig. 4g), providing further evidence that TAU^P301L^ expression fails to elicit a neurodegeneration-regeneration cascade in adult zebrafish brain. Since zebrafish brain is highly regenerative after various type of neuronal loss^33,45,67,79^, we believe that the lack of stem cell proliferation response and activation of regenerative programs is an indication that adult zebrafish brain can cope well with the phosphorylated forms of TAU^P301L^.

Several studies documented Tauopathy-related neurodegeneration phenotypes in the absence of tangle formation^17,18,80–82^. Conflicting with these findings, Tau aggregation was also proposed as the major cause of toxicity in animal models^1,2,4,9,13,56,61,83^. We have to note that in our genetic model, we have used the longest isoform of human TAU protein (2N4R). Different versions of TAU have been shown to have different levels of aggregation potential, and therefore, in future other human TAU versions could be tested in zebrafish to come to a more consolidated conclusion of a protective mechanism preventing tangle formation. Additionally, protein aggregation is temperature dependent, and zebrafish lives at 28 °C while our body temperature is 37 °C. Therefore, a possible link between the temperature and aggregation dynamics of TAU could also account for the observed differences, and this aspect of Tauopathies can also be studied in zebrafish. Given that keeping zebrafish at 37 °C has been used as a means of heat stress, the effects of temperature on folding, phosphorylation and aggregation dynamics and clearance of TAU are therefore interesting aspects of TAU toxicity that can be tested *in vivo* in zebrafish.

In overall, we believe that our new chronic conditional TAU expression model in zebrafish is a useful assay system where various neurodegeneration paradigms could be modularly combined, and the effects of any intervention on the TAU aggregation could be measured. For instance, our model would allow screening for molecules or pathways that could abrogate a putative “protective mechanism” that prevents phosphorylated TAU from getting aggregated. In such a case, adult zebrafish brain may potentially start generating neurofibrillary tangles. Alternatively, possible factors that are related to immune system could have effects on how TAU is phosphorylated and how phosphorylated forms of TAU proceed further to form oligomers and neurofibrillary tangles. Such “reverse” studies could uncover currently unknown mechanisms that may help us to further understand and design therapies for Tauopathies in humans.

Zebrafish brain is highly regenerative and responds to injuries by activating specific molecular programs^33,45,67^. A possible regenerative therapy in human brains could entail mobilizing the endogenous stem cells to form new neurons that replace the lost ones, and regulation of the synaptic plasticity to compensate for the lost circuit connections. Reductionist models of neurodegenerative diseases in zebrafish can therefore help us to understand which types pathogenicity would activate regenerative programs in stem cells. By cataloging the effects of individual pathogenic hallmarks of neurodegenerative diseases in zebrafish, this animal model can help clinical studies to design regenerative therapies or prevention strategies that are naturally occurring in a vertebrate brain. Since previous zebrafish models of Tauopathies did not focus on the regenerative response of the brain, our model is also a useful addition to the toolbox of regeneration research in order to understand how a vertebrate brain responds to toxic protein aggregation or even prevents the progression of disease pathology.

## Materials and Methods

### Ethics statement

All animal experiments were carried out in accordance with the animal experimentation permits of Referate 24 (Veterinärwesen, Pharmazie, und GMP) of the state administration office of Saxony, Germany (Landesdirektion Sachsen) and the ethical commission of TU Dresden (Kommission für Tierversuche). The approved experimental protocols are licensed under the permit numbers TVV-35/2016 and TVV-52/2015 through the regular official processes.

### Generation of transgenic zebrafish lines and recombination

The longest isoform of human TAU (2N4R) with P301L mutation template was a gift from Bettina Schmid and Christian Haass^37^. The template was amplified by PCR using the forward (5′-atggctgagccccgccag-3′) and reverse (5′-tcacaaaccctgcttggccagg-3′) primer pairs that were flanked by *XhoI* and *AscI* restriction enzymes, respectively. The PCR product was subcloned into pGEM-T-Easy vector (Promega) and sequenced by M13 universal primers. The TAU^P301L^ was released from pGEM-T-Easy by *XhoI/AscI* enzymes and cloned into pTol(Red-to-Green) (ubiquitin promoter-driven^72^ loxP-DsRed-loxP-GFP cassette-containing Tol2 transgenesis vector^47,84^). For generating neural beta tubulin (nbt) promoter-driven cre line, we amplified the *nbt* promoter using forward (5′-tgggccctctagaccctgtctg-3′) and reverse (5′-ggccggccgattgggttgagtc-3′) primers flanked by *ApaI* and *FseI* restriction enzyme sites. PCR product was subcloned into *her4*.*1*:mCherry-t2a-Cre^ERT2^ transgenesis plasmid containing Tol2 sites^84,85^ by using appropriate restriction enzymes.

For transgenesis, we used a protocol as described^85^ with minor modifications. Briefly, 1 nL of 30 ng/µL plasmid and Tol2 transposase mix in RNAse-free water were injected into fertilized zebrafish eggs (F0), the fish were raised to adulthood, and were outcrossed to wild type zebrafish (AB background) to establish the founder fish (F1). 6 founders were identified, and were raised to adulthood for Tg(ubi:loxp-DsRed-loxp-GFP-t2a-hTAUP301L). Tg(nbt:mCherry-t2a-Cre^ERT2^) founders were identified by outcrossing to wild type animals and screening for mCherry expression. Positive embryos were raised to adulthood and crossed to a control cre-effector line that shows fluorescence after recombination in order to test the effective recombination capacity. To identify a cre-effector TAU line with high efficiency of recombination, all F1 fish for TAU were outcrossed to Tg(her4.1:mCherry-t2a-Cre^ERT2^) or Tg(nbt:mCherry-t2a-Cre^ERT2^) cre-driver lines, and the F2 progeny were treated with freshly prepared 5 µM of Tamoxifen (TAM) in E3 medium between 16–32 hpf and 32–48 hpf. At 48hpf, the F2 progeny with high GFP expression were sorted from non-GFP siblings. The outcrosses were repeated to obtain a stably segregating transgene insertion (achieved after F4). The experiments and results given here are from F5 and later offspring.

### Tissue preparation and immunohistochemical stainings

The fish were sacrificed, and the heads were fixed in 2% PFA overnight at 4 °C. After washing 3 times 10 min with Phosphate buffer, the heads were decalcified using 20% Sucrose/EDTA. Samples were mounted in 20% Sucrose/7.5% Gelatin as described^39^. Samples were cryosectioned at 12 µM thickness, and were stained with the following antibodies: chicken anti-GFP IgG (1:2000; Abcam), mouse anti-Tau13 IgG1 (IHC 1:1000, WB 1:500; Abcam), mouse anti-phospho-Tau (AT180) IgG1 (IHC 1:500, WB 1:1000; Pierce, Thermo Scientific), mouse anti-phospho-Tau (AT8) IgG1 (IHC 1:500, WB 1:1000; Pierce, Thermo Scientific), mouse anti-phospho-Tau (AT270) IgG1 (IHC 1:500, WB 1:1000; Pierce, Thermo Scientific), mouse anti-PCNA IgG2a (1:500; Dako), rabbit anti-S100β IgG (1:500; Dako), rabbit-anti-L-plastin IgG (1:5000) (gift from Michael Redd), rabbit anti-beta actin (WB 1:1000, Abcam), rabbit anti-synaptophysin (1:500, Abcam), mouse anti-HuC/D IgG2b (1:500, Life Technologies), mouse anti IL4 (1:500, R&D Systems), and rabbit anti-Tau (T22) (1:500, Merck).

### Gallyas silver staining

Brain samples were fixed as described above. Heads were embedded in paraffin, sectioned at a thickness of 10 µM. Samples were pretreated with 0.25% potassium permanganate at room temperature for 15 min^86^, washed in 2% oxalic acid for 2 min, and incubated in 0.4% lanthanum nitrate/2% sodium acetate/3% H_2_O_2_ solution at room temperature for 60 min^87^. The following treatments were performed: 5% periodic acid (5 min), alkaline silver iodide solution (1 min), 0.5% acetic acid (10 min), developer solution containing tungsto-silicic acid (5–10 min), 0.5% acetic acid, 0.1% gold chloride (5 min), and 1% sodium thiosulfate (5 min). Samples were counterstained with 0.1% nuclear fast red (2 min), dehydrated using Methanol, and were mounted in DPX.

### Western blotting

For western blotting, the telencephalon of 6 month-old fish were dissected or 9 day-old larvae were sacrificed according the local ethical regulations. The telencephalon or whole larvae were used for protein isolation using 200 µL RIPA buffer (Sigma, R0278) with addition of phosphatase (Roche, Catalog Number 04906837001) and protease inhibitors (Roche, Catalog number 04963132001). 5 µl of protein ladder (ThermoFisher, #26634) and 10 µl of total protein was loaded in 4–12% Bis-Tris precast gradient gels (NuPage, Catalog number NP0322BOX) and the gels were run in NuPAGE MES SDS running buffer (Novex, Life technologies, NP0006-1) at 200 V for 60 min. Blots were transferred to methanol activated PVDF (Novex, Life technologies, LC2002) membrane, and were blocked in 10% milk powder in 0.2% Tween in 1X-PBS for 1h at room temperature. The primary antibodies at appropriate dilutions in 2 ml 0.2% Tween in 1X-PBS were applied overnight at 4 °C in 15 or 50 ml plastic containers. Following the washing steps, secondary antibodies at 1:4000 dilutions (anti-rabbit IgG HRP (Santa Cruz, #sc-2004) or anti-mouse IgG peroxidase (Sigma, #A8924)) were applied at room temperature for 2 h. Gel images were acquired by ImageQuant LAS4000 (GE Healthcare) using Western BLoT Ultra Sensitive HRP Substrate (Takara, #T7104A).

### Cerebroventricular microinjection

Zebrafish at the age of 6 months were injected with 20 µM Aβ42 as described previously^39,88–90^. For cerebroventricular microinjection, the fish were first anaesthetized in 0.001% MESAB, and a slit was generated in the skull over the optic tectum without damaging the brain. 1 µl of solution containing monomeric Amyloid-β42^39,40^ was injected into the cerebroventricular fluid (for control animals PBS was injected). The fish were returned to their containers and recovery was carefully observed. All the injected fish survived the procedure and did not show any defects that might be due to the injections. 25 µg of Okadaic acid (Sigma #O8010) was dissolved in 500 µl distilled 1x-PBS, and 5 ng/µL was injected into the fish brain as described previously^89,90^. 3 dpi fish were sacrificed and prepared for immunohistochemical stainings as described above.

### Detection of mRNA expression levels

mRNA isolation and detection of relative mRNA expression levels were performed as described^39,40^.

### Imaging, quantification, and statistical analyses

Images were acquired using structured illumination microscope (Zeiss AxioImager Z1) and scanning confocal microscope (Leica SP5 II, DM6000). In order to maintain the comparative intensities of the images and not to introduce bias, we used the same settings for all experimental groups of controls and transgenic animals per experiment. Images were taken using the tiling option of the Apotome microscope with no auto-correction to prevent inconsistencies in fluorescence intensity. Acquired images were stitched together using the Zeiss ZEN software installed to the Apotome configuration. Raw images are used to generate figures using Adobe Photoshop and Adobe Illustrator software. Cell counting was performed manually on the entire sections taken on a slide and stereological calculations were performed as described^91^. The statistical analyses were performed using GraphPad Prism (Version 6.02) for one-way ANOVA followed by a Tukey’s post-hoc test and for Student’s T-Test. Error bars shown are the s.d, and asterisks indicate significance: *p < 0.05, **p < 0.01, ***p < 0.001. p > 0.05 is not significant (n.s.). Student’s T-test was performed for paired samples, and a T-Test for independent measurements. All sample sizes and analyses powers were calculated using G*Power-Software, (http://www.gpower.hhu.de/en.html). No animals were excluded from the analyses. n = 5 fish for every experimental group, and in total more than 20 sections were used for stereological quantification. The ages of the animals used are denoted in the respective figure legends. Quantification of band intensities in western blots were performed as described^92^.

### Behavioral analyses

Escape response assays were performed as described^37^. Tamoxifen was replaced with fresh E3 medium. The video and imaging taken by changing filter from bright field to DsRED and then to GFP. The escape response was recorded at GFP channel to localize the dTg fish. Escape response was measured by touching the tail using Olympus MVX10 stereo microscope and CellSens Dimension software.

### Data Availability

All data generated or analyzed during this study are included in this published article (and its Supplementary Information files).

## Electronic supplementary material

Supplementary Information

Supplementary Video 1

Supplementary Video 2
