# Supplementary material for: Human TAUP301L overexpression results in TAU hyperphosphorylation without neurofibrillary tangles in adult zebrafish brain
Source: Sci Rep. 2017 Oct 11;7:12959. doi: 10.1038/s41598-017-13311-5 (PMC5636889; doi:10.1038/s41598-017-13311-5)
Supplement: Supplementary file 1 — Supplementary Information [file 41598_2017_13311_MOESM1_ESM.pdf]

## Supplementary Information

# Human TAU$^{P301L}$ overexpression results in TAU hyperphosphorylation without neurofibrillary tangles in adult zebrafish brain

Mehmet I Cosacak[1], Prabesh Bhattarai[1], Ledio Bocova[1], Tim Dzewas[1], Violeta Mashkaryan[1,2], Christos Papadimitriou[1], Kerstin Brandt[1], Heike Hollak[1], Christopher L Antos[3], Caghan Kizil[1,2,*]

1 German Center for Neurodegenerative Diseases (DZNE), Arnoldstrasse 18, 01307, Dresden, Germany.

2 Center for Regenerative Therapies Dresden (CRTD), TU Dresden, Fetscherstrasse 105, 01307, Dresden, Germany.

3 School of Life Sciences and Technology, ShanghaiTech University, 100 Haike Road, Shanghai, China.

* Corresponding author: C.K.: caghan.kizil@dzne.de

## Supplementary Figures and legends

## Supplementary Figure 1

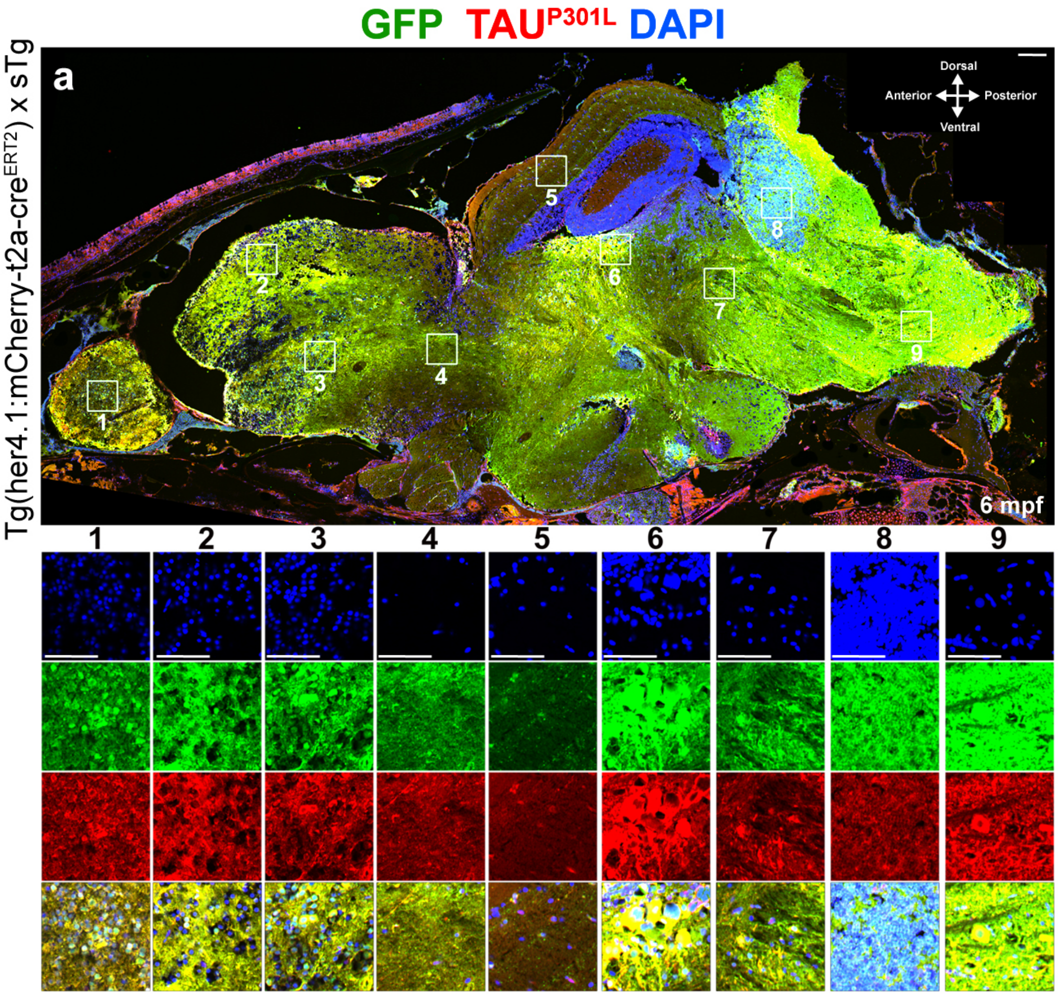


Supplementary Fig. 1: (a) Immunohistochemical staining for GFP and TAU$^{P301L}$ on a sagittal section of a 6 month-old dTg animal. Insets from 1 to 9 are DAPI (blue), GFP (green), TAU$^{P301L}$ (red), and merged images from olfactory bulb (1), pallium (2), subpallium (3), midbrain (4), optic tectum (5), medial longitudinal fascicle (6), tegmentum (7), cerebellum (8) and medulla oblongata (9). Scale bars equal 100 μm. n = 8 fish.

**Supplementary Figure 2**

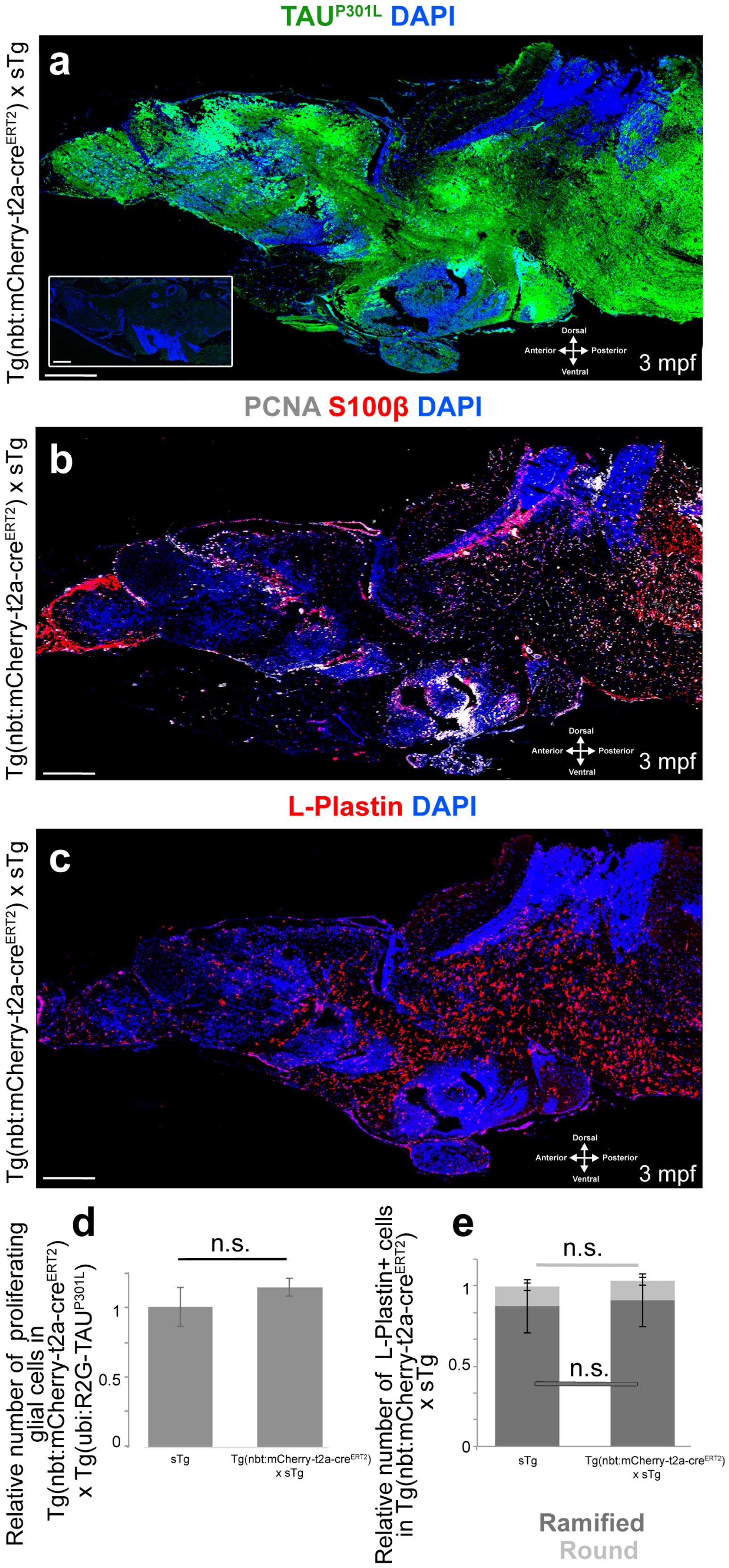

Supplementary Fig. 2. (a) Immunohistochemical staining for $TAU^{P301L}$ on a sagittal section of a 3 month-old Tg(nbt:mCherry-t2a-creERT2) x Tg (ubi:loxP-DsRed-loxP-GFP-t2a-$TAU^{P301L}$) animal recombined as in Fig. 1. Inset shows the unrecombined animal. (b) Immunohistochemical staining for PCNA and S100β on a serial section of the same animal. (c) Immunohistochemical staining for L-Plastin on a serial section of the same animal. (d) Comparative quantification of the number of proliferating glial cells. (e) Comparative quantification of the relative numbers of L-Plastin-postive cells. Values represent mean ± s.e.m. n.s.: not significant. Scale bars equal 100 μm.

**Supplementary Figure 3**

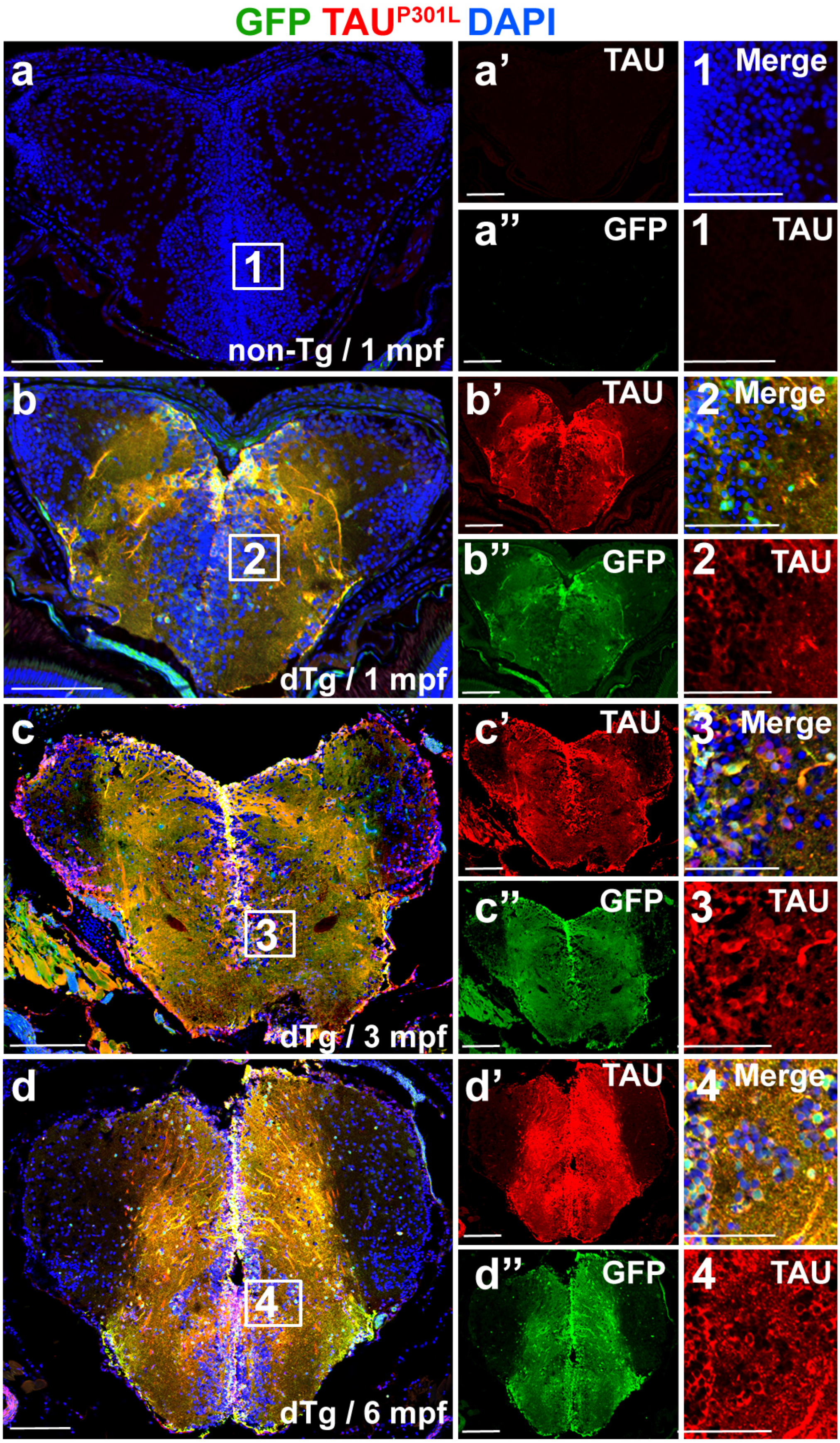

Supplementary Fig. 3: (a) Immunohistochemistry (IHC) for $TAU^{P301L}$ (red) and GFP (green) on coronal sections of telencephalon of a 1 month-old non-transgenic animal. (a', a'') Individual fluorescent channels for $TAU^{P301L}$ (a') and GFP (a''). (1) The enlarged view of the inset in a. (b) IHC for $TAU^{P301L}$ and GFP on coronal sections of telencephalon of a 1-month old dTg animal. (b', b'') Individual fluorescent channels for $TAU^{P301L}$ (b') and GFP (b''). (2) The enlarged view of the inset in b. (c) IHC for $TAU^{P301L}$ and GFP on coronal sections of telencephalon of a 3-month old dTg animal. (c', c'') Individual fluorescent channels for $TAU^{P301L}$ (c') and GFP (c''). (3) The enlarged view of the inset in c. (d) IHC for $TAU^{P301L}$ and GFP on coronal sections of telencephalon of a 6-month old dTg animal. (d', d'') Individual fluorescent channels for $TAU^{P301L}$ (d') and GFP (d''). (4) The enlarged view of the inset in d. Scale bars equal 50 µm. n = 4 fish and >25 histological sections for every staining.

**Supplementary Figure 4**

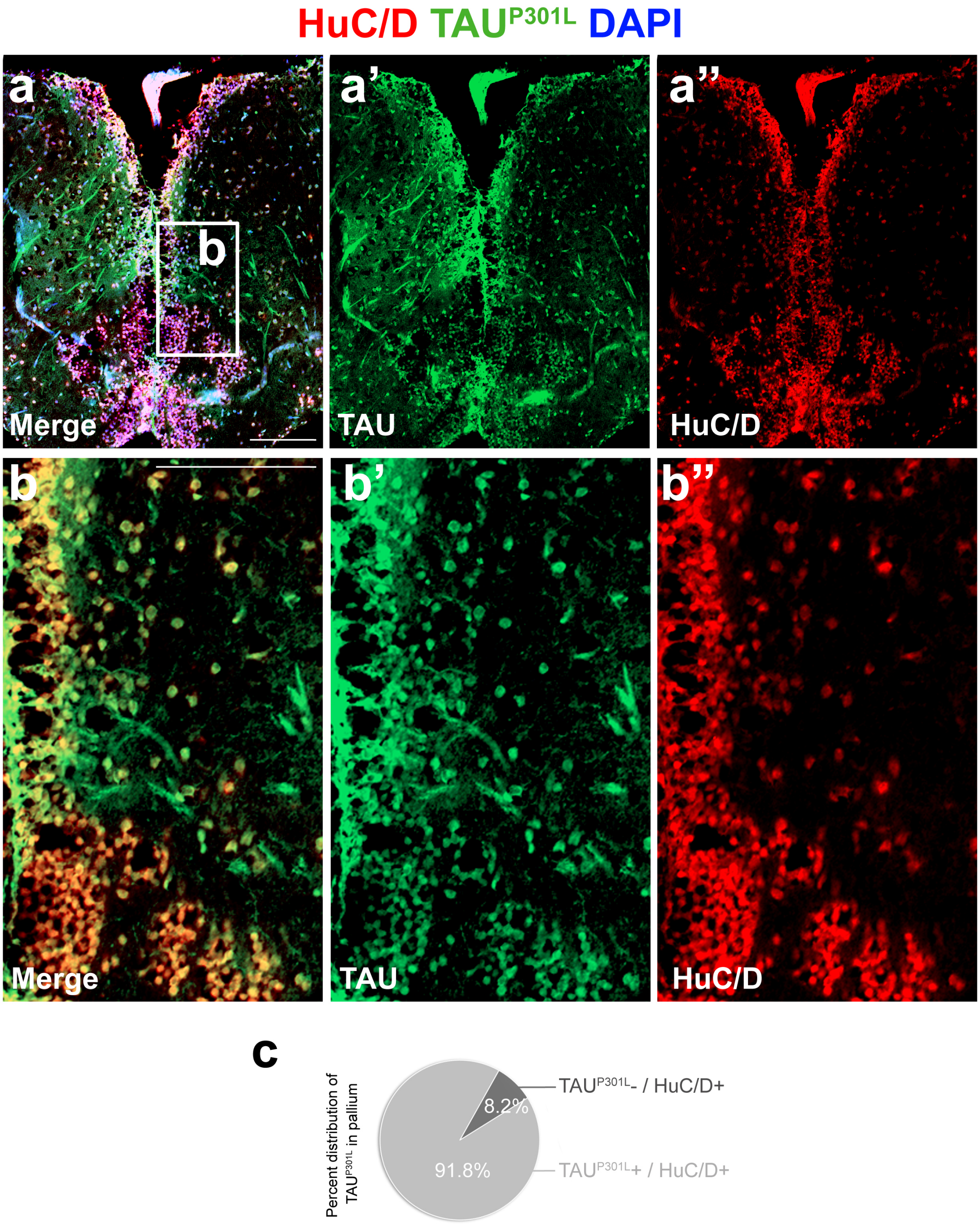


Supplementary Fig. 4: Immunohistochemistry for HuC/D (red) and TAU$^{P301L}$ (green) on the telencephalon of a 6 month-old dTg animal. (a', a'') Individual channels for green and red. (b-b'') High-magnification image of the frame in a. (c) Quantification of the percentage of HuC/D-positive neurons expressing TAU in the pallium. Scale bars equal 100 μm. n = 4 fish and >20 histological sections for every staining and quantification.

**Supplementary Figure 5**

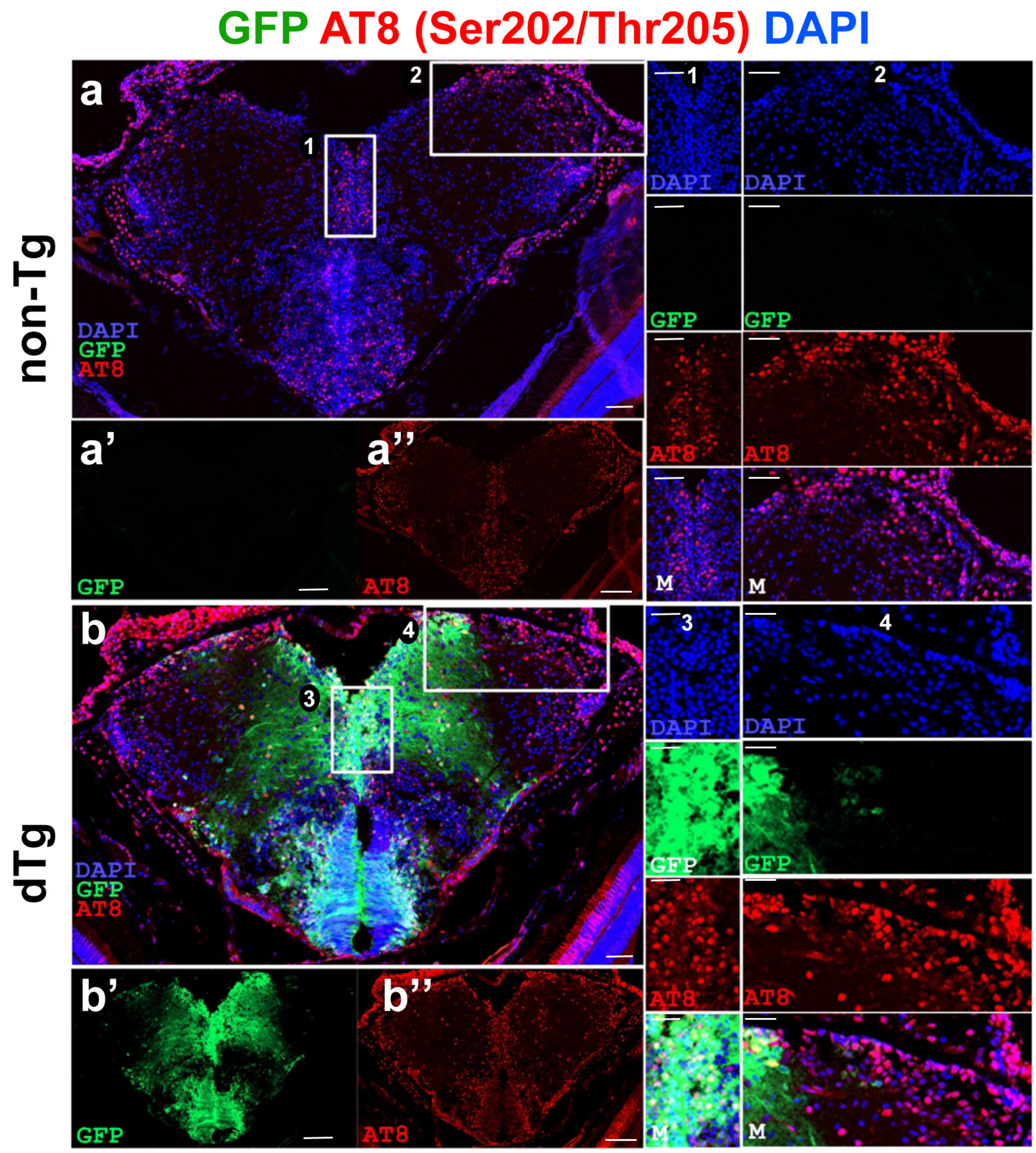


Supplementary Fig. 5: (a) Immunohistochemistry (IHC) for AT8 (red) and GFP (green) on coronal sections of telencephalon of a 6 month-old non-transgenic animal. (a', a'') Individual fluorescent channels for GFP (a') and AT8 (a''). (1 and 2) The enlarged view of the inset in A with individual channels for DAPI, GFP and AT8, and merged image. (b) IHC for AT8 and GFP on coronal sections of telencephalon of a 6-month old dTg animal. (b', b'') Individual fluorescent channels for GFP (b') and AT8 (b''). (3 and 4) The enlarged view of the inset in b with individual channels for DAPI, GFP and AT8, and merged image. Scale bars equal 25 μm. n = 5 fish for every staining.

**Supplementary Figure 6**

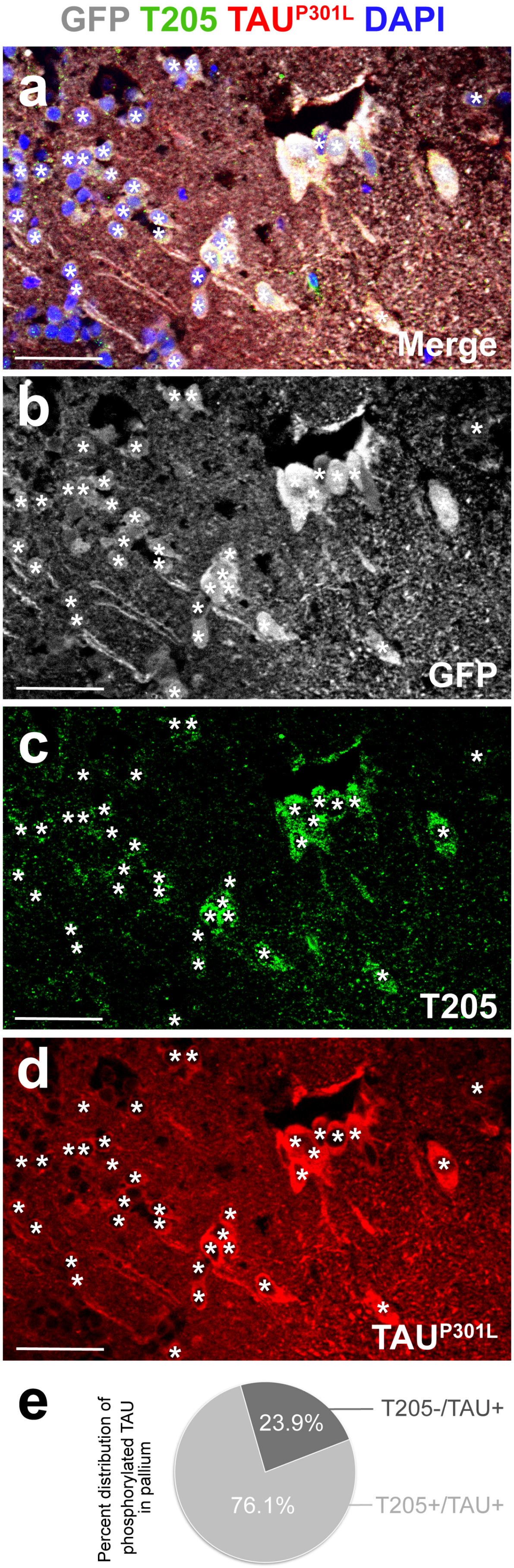
GFP T205 TAU^P301L DAPI
a
Merge
b
GFP
c
T205
d
TAU^P301L
e
Percent distribution of phosphorylated TAU in pallium
23.9%
T205-/TAU+
76.1%
T205+/TAU+

Supplementary Fig. 6: Immunohistochemical staining for GFP (recombined cells, white), T205 (hyperphosphorylated TAU, green), and $TAU^{P301L}$ (red) in 6 month-old dTg animal. (a) merge image. Individual channels for (b) GFP, (c) T205, and (d) $TAU^{P301L}$. (e) Quantification of the percentage of $TAU^{P301L}$-positive cells that are T205 positive or negative. Scale bars equal 50 μm. n = 3 fish and >20 histological sections for every staining and quantification.

**Supplementary Figure 7**

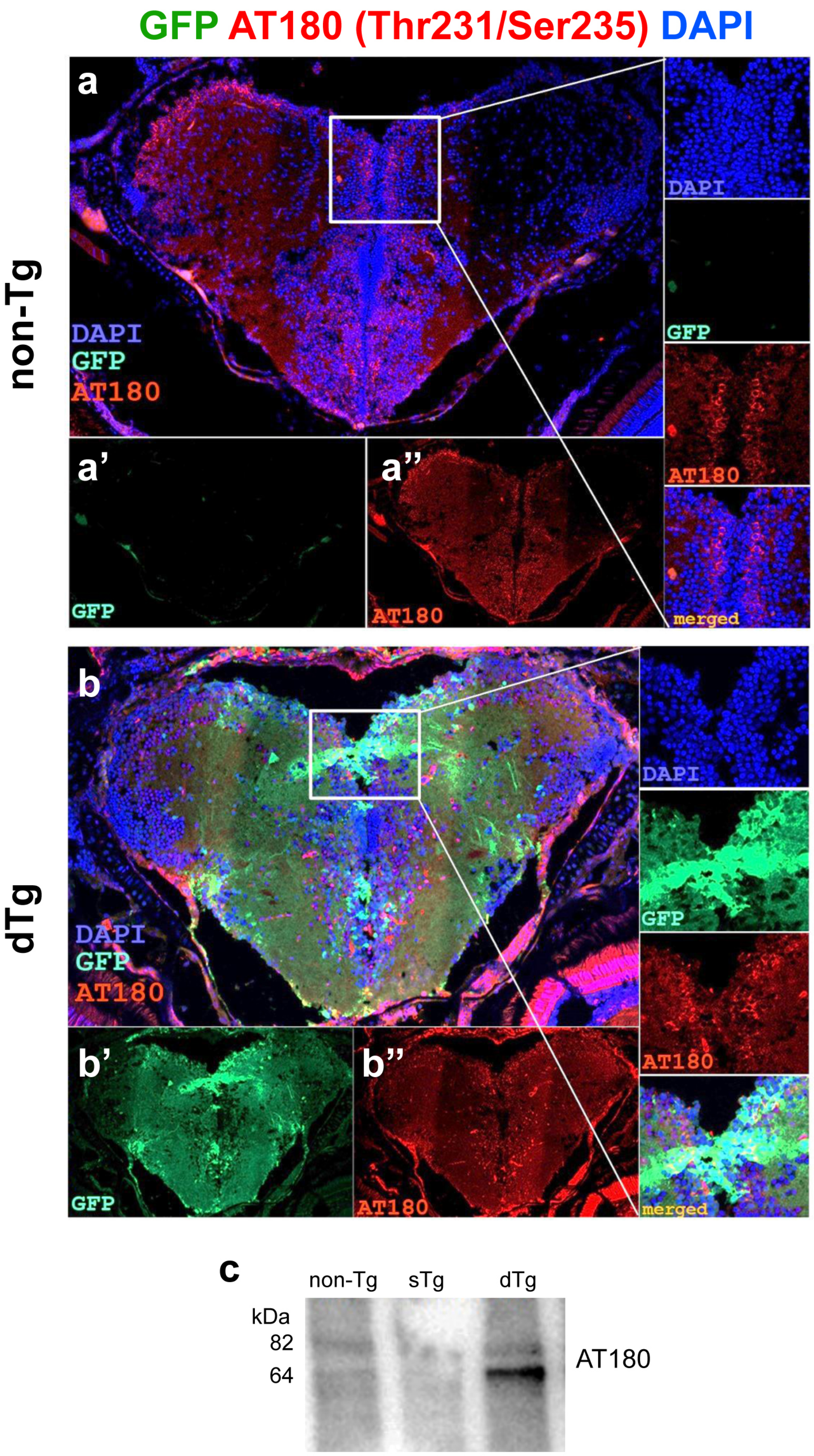

Supplementary Fig. 7: (a) Immunohistochemistry (IHC) for AT180 (red) and GFP (green) on coronal sections of telencephalon of a 6-month old non-transgenic animal. (a’, a’’) Individual fluorescent channels for GFP (a’) and AT180 (a’’). Insets show the enlarged view of the frame in A with individual channels for DAPI, GFP and AT180, and merged image. (b) IHC for AT180 and GFP on coronal sections of telencephalon of a 6-month old dTg animal. (b’, b’’) Individual fluorescent channels for GFP (b’) and AT180 (b’’). Insets show the enlarged view of the frame in b with individual channels for DAPI, GFP and AT180, and merged image. (c) Western blot for AT180 from brains of non-Tg, sTg and dTg animals. Scale bars equal 25 µm. n = 5 fish for every staining.

**Supplementary Figure 8**

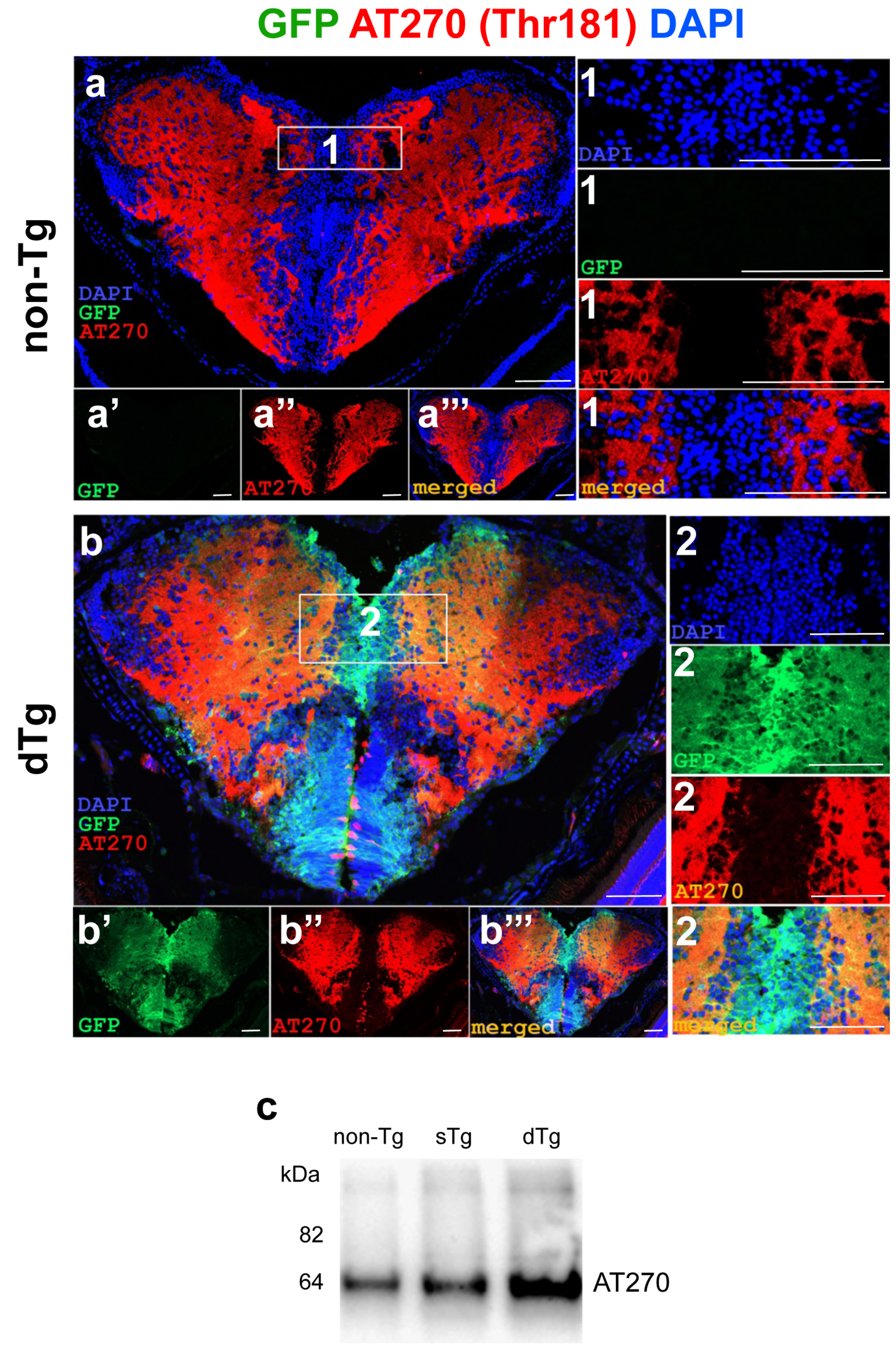

Supplementary Fig. 8: (a) Immunohistochemistry (IHC) for AT270 (red) and GFP (green) on coronal sections of telencephalon of a 6-month old non-transgenic animal. (a’, a’’) Individual fluorescent channels for GFP (a’) and AT270 (a’’). Insets show the enlarged view of the frame in a with individual channels for DAPI, GFP and AT270, and merged image. (b) IHC for AT270 and GFP on coronal sections of telencephalon of a 6-month old dTg animal. (b’, b’’) Individual fluorescent channels for GFP (b’) and AT270 (b’’). Insets show the enlarged view of the frame in b with individual channels for DAPI, GFP and AT270, and merged image. (c) Western blot for AT270 from brains of non-Tg, sTg and dTg animals. Scale bars equal 50 μm. n = 6 fish for every staining.

**Supplementary Figure 9**

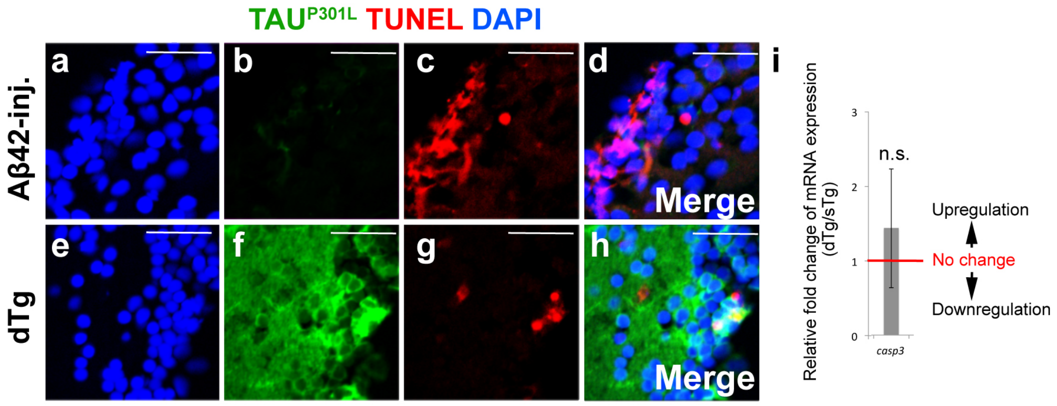


Supplementary Fig. 9: (a-d) Immunohistochemistry (IHC) for TAU$^{P301L}$ (green) and staining for apoptotic cells with TUNEL (green) telencephalon of a 6-month old non-transgenic animal injected with Amyloid-beta42. (a-c) Individual fluorescent channels for DAPI (a), TAU$^{P301L}$ (b), and TUNEL (c). (d) Merged image. (e-h) IHC for TAU$^{P301L}$ (green) and staining for apoptotic cells with TUNEL (green) telencephalon of a 6-month old dTg animal. (a-c) Individual fluorescent channels for DAPI (e), TAU$^{P301L}$ (f), and TUNEL (g). (h) Merged image. (i) Quantification of relative change in the expression levels of *casp3* mRNA in dTg compared to sTg adult zebrafish brains. Scale bars equal 25 µm. n = 5 fish for every staining.

**Supplementary Figure 10**

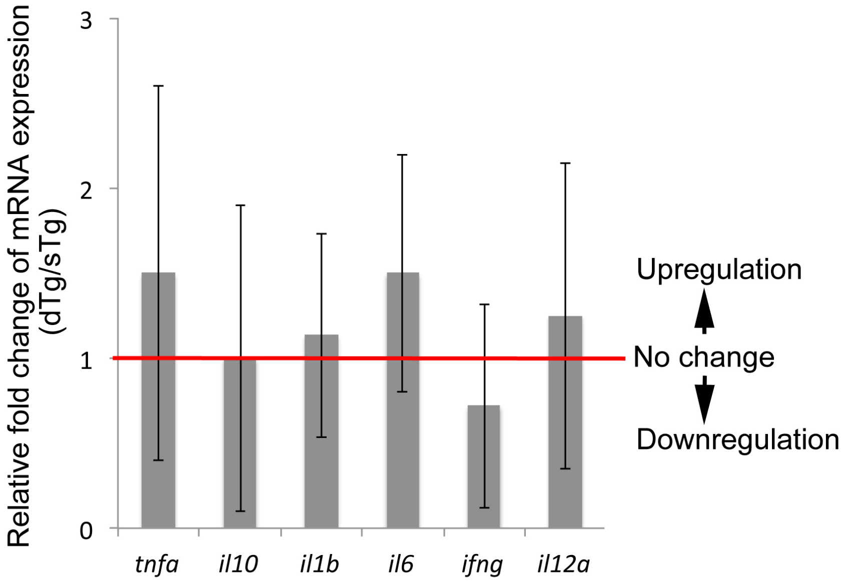


Supplementary Fig. 10: Quantification of the relative change in the expression levels of proinflammatory in dTg compared to sTg adult zebrafish brains.

## Supplementary Figure 11

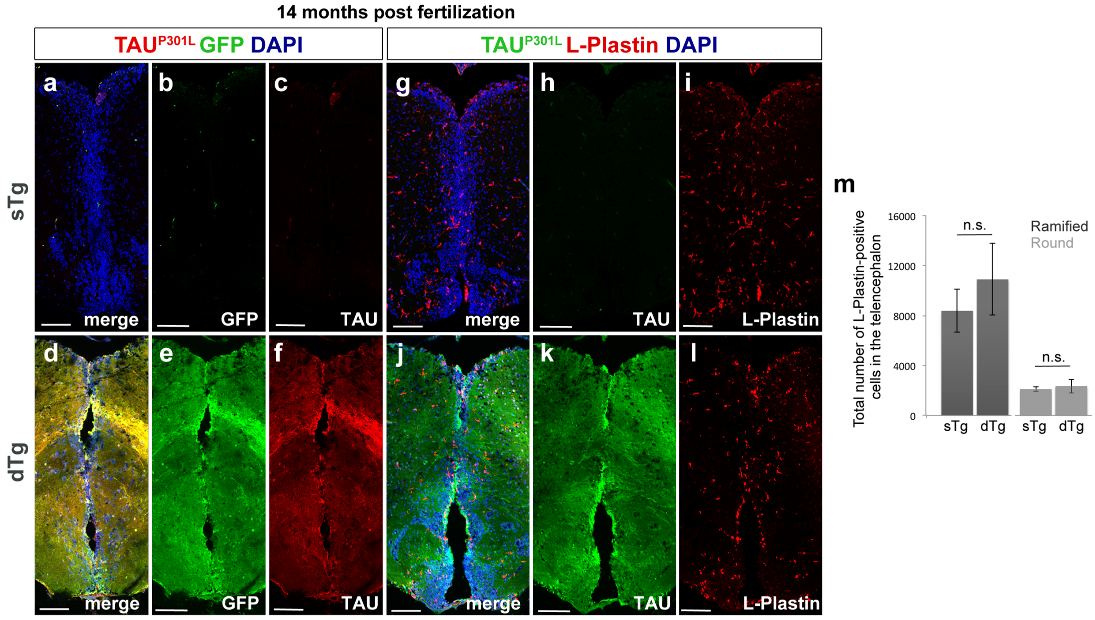


Supplementary Fig. 11: (a) Immunohistochemistry (IHC) for TAU$^{P301L}$ (red) and GFP (green) on coronal sections of telencephalon of a 14-month old sTg animal. (b,c) Individual fluorescent channels for TAU$^{P301L}$ (b) and GFP (c). (d) IHC for TAU$^{P301L}$ and GFP on coronal sections of telencephalon of a 14 month-old dTg animal. (e,f) Individual fluorescent channels for TAU$^{P301L}$ (e) and GFP (f). (g) IHC for L-Plastin and TAU$^{P301L}$ on coronal sections of telencephalon of a 14 month-old sTg animal. (h,i) Individual fluorescent channels for TAU$^{P301L}$ (h) and L-Plastin (i). (j) IHC for L-Plastin and TAU$^{P301L}$ on coronal sections of telencephalon of a 14-month old dTg animal. (k,l) Individual fluorescent channels for TAU$^{P301L}$ (k) and L-Plastin (l). (m) Quantification of round and ramified L-Plastin-positive cells in the telencephalon of 14-month-old sTg and dTg animals. Values represent mean ± s.e.m. *: $p<0.05$, **: $p<0.01$, ***: $p<0.005$. Scale bars equal 50 µm. n = 5 fish and >25 histological sections for every staining.

**Supplementary Figure 12**

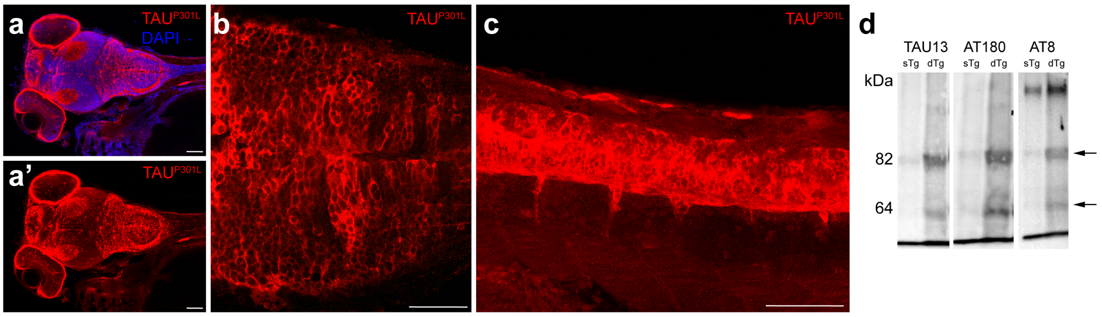


Supplementary Fig. 12: (a) Immunohistochemical staining for TAU$^{P301L}$ in 9 day-old dTg larvae. Anterior view of the brain is shown. (b) Single fluorescence channel for TAU$^{P301L}$. (c) High-magnification image of the frame in b. (d) TAU$^{P301L}$ expression in the spinal cord. (e) Western blot analyses of TAU$^{P301L}$ (TAU13), and hyperphosphorylated form of TAU with AT180 and AT8. Scale bars equal 50 µm.

**Supplementary Figure 13**

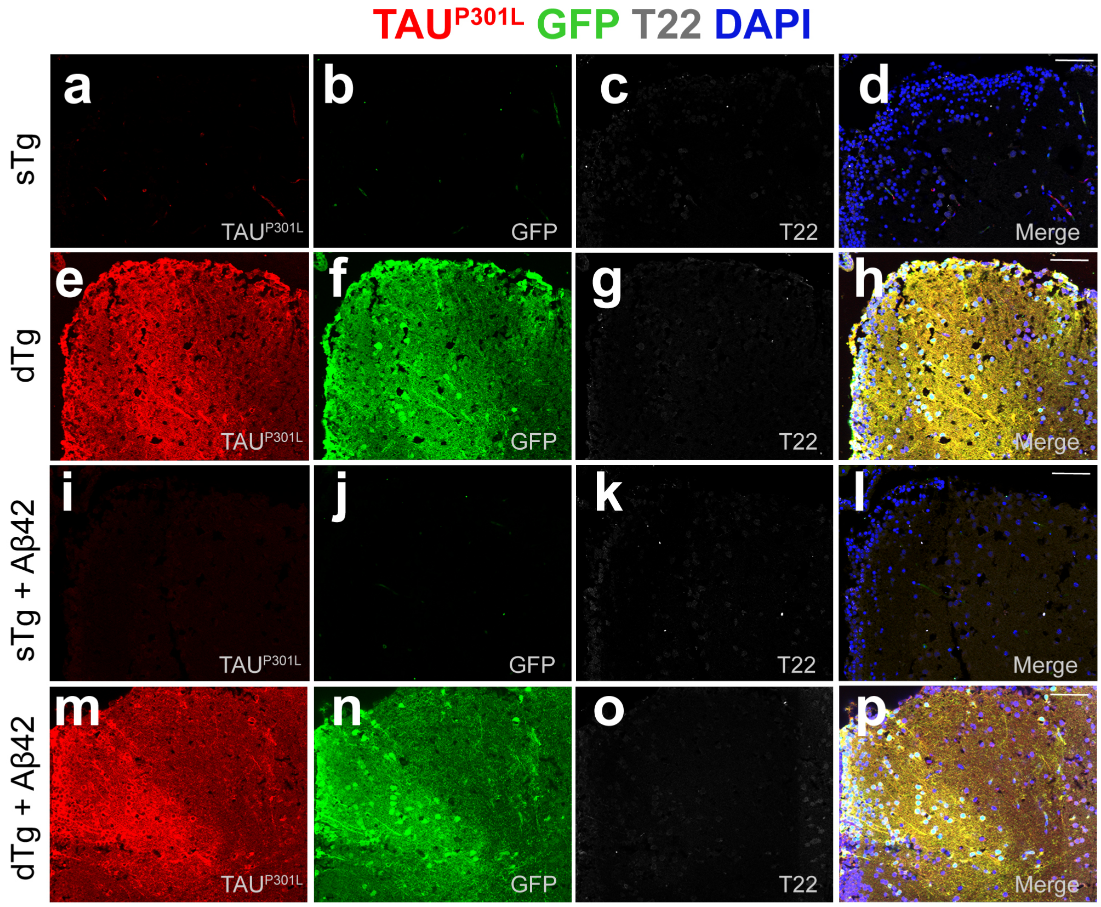


Supplementary Fig. 13: Immunohistochemical staining for TAU$^{P301L}$ (red), GFP (green), and T22 (TAU oligomers, gray) in sTg (a-d), dTg (e-h), sTg injected with Aβ42 (i-l), and dTg injected with Aβ42 (m-p). Scale bars equal 50 µm. All animals are 6 month-old.

## Supplementary Video Captions

Supplementary Video 1: Escape response assay for 2 day-old dTg animals.

Supplementary Video 2: Escape response assay for 4 day-old non-transgenic, sTg and dTg animals for *her4.1* or *nbt* promoter-driven conditional TAU transgenic lines of zebrafish.
